# Attenuating mitochondrial dysfunction and morphological disruption with PT320 delays dopamine degeneration in MitoPark mice

**DOI:** 10.1186/s12929-024-01025-6

**Published:** 2024-04-17

**Authors:** Vicki Wang, Kuan-Yin Tseng, Tung-Tai Kuo, Eagle Yi-Kung Huang, Kuo-Lun Lan, Zi-Rong Chen, Kuo-Hsing Ma, Nigel H. Greig, Jin Jung, Ho-II Choi, Lars Olson, Barry J. Hoffer, Yuan-Hao Chen

**Affiliations:** 1 Doctoral Degree Program in Translational Medicine, National Defense Medical Center and Academia Sinica, Taipei, 11490 Taiwan; 2 Graduate Institute of Medical Sciences, National Defense Medical Center, Taipei, 11490 Taiwan; 3 Department of Neurological Surgery, Tri-Service General Hospital, Taipei, 11490 Taiwan; 4 National Defense Medical Center, Taipei, 11490 Taiwan; 5 Department of Pharmacology, National Defense Medical Center, Taipei, 11490 Taiwan; 6 Department of Pathology, Tri-Service General Hospital, Taipei, 11490 Taiwan; 7 Graduate Institute of Biology and Anatomy, National Defense Medical Center, Taipei, 11490 Taiwan; 8 Drug Design & Development Section, Translational Gerontology Branch, Intramural Research Program National Institute on Aging, National Institutes of Health (NIH), Baltimore, MD 21224 USA; 9 Peptron, Inc., Yuseong-gu, Daejeon, 34054 Republic of Korea; 10 Department of Neuroscience, Karolinska Institute, 171 77 Stockholm, Sweden; 11 Department of Neurosurgery, University Hospitals of Cleveland, Case Western Reserve University School of Medicine, Cleveland, OH 44106 USA

**Keywords:** Glucagon-like peptide-1 receptor agonist, MitoPark mouse, Parkinson’s disease, Exenatide, Mitochondria, PT320

## Abstract

**Background:**

Mitochondria are essential organelles involved in cellular energy production. Changes in mitochondrial function can lead to dysfunction and cell death in aging and age-related disorders. Recent research suggests that mitochondrial dysfunction is closely linked to neurodegenerative diseases. Glucagon-like peptide-1 receptor (GLP-1R) agonist has gained interest as a potential treatment for Parkinson's disease (PD). However, the exact mechanisms responsible for the therapeutic effects of GLP-1R-related agonists are not yet fully understood.

**Methods:**

In this study, we explores the effects of early treatment with PT320, a sustained release formulation of the GLP-1R agonist Exenatide, on mitochondrial functions and morphology in a progressive PD mouse model, the MitoPark (MP) mouse.

**Results:**

Our findings demonstrate that administration of a clinically translatable dose of PT320 ameliorates the reduction in tyrosine hydroxylase expression, lowers reactive oxygen species (ROS) levels, and inhibits mitochondrial cytochrome c release during nigrostriatal dopaminergic denervation in MP mice. PT320 treatment significantly preserved mitochondrial function and morphology but did not influence the reduction in mitochondria numbers during PD progression in MP mice. Genetic analysis indicated that the cytoprotective effect of PT320 is attributed to a reduction in the expression of mitochondrial fission protein 1 (Fis1) and an increase in the expression of optic atrophy type 1 (Opa1), which is known to play a role in maintaining mitochondrial homeostasis and decreasing cytochrome c release through remodeling of the cristae.

**Conclusion:**

Our findings suggest that the early administration of PT320 shows potential as a neuroprotective treatment for PD, as it can preserve mitochondrial function. Through enhancing mitochondrial health by regulating Opa1 and Fis1, PT320 presents a new neuroprotective therapy in PD.

**Graphical Abstract:**

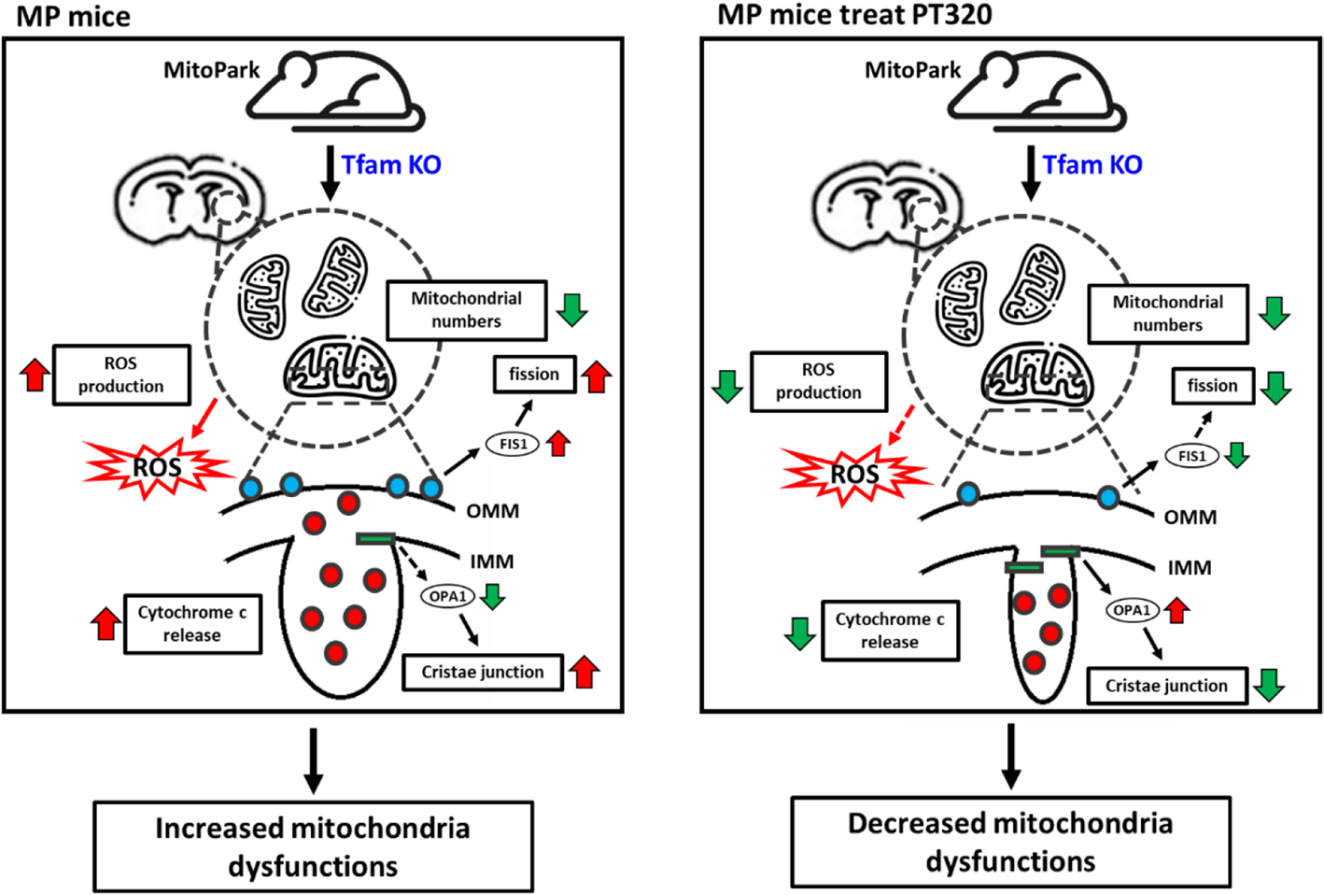

**Supplementary Information:**

The online version contains supplementary material available at 10.1186/s12929-024-01025-6.

## Background

Parkinson's Disease (PD) is the second most common progressive neurodegenerative disorder [1]. The disease is characterized by the gradual loss of dopamine-producing neurons in the substantia nigra pars compacta (SNc), which forms the nigrostriatal dopaminergic pathway. This leads to a severe deficiency of dopamine in the putamen and caudate nucleus, resulting in the loss of motor coordination as well as other symptoms. Current treatments for PD primarily involve pharmacological replacement of dopamine to alleviate motor symptoms, providing symptomatic relief in the early stages. However, these treatments do not halt or reverse neuron loss, nor do they modify the disease's progression. PD is believed to result from a combination of factors, including oxidative stress, mitochondrial dysfunction, protein mishandling, and genetic predisposition [2], but the exact causes remain unknown.

Mitochondria play a crucial role in cellular energy production, and their dysfunction is well-established as a contributing factor in the pathogenesis of PD [3–5]. This dysfunction includes the loss of electron transport chain (ETC) protein expression and activity within the mitochondria. The ETC is not only responsible for ATP production, which is essential for cellular energy, but it also generates endogenous reactive oxygen species (ROS) as a byproduct. In PD, inhibition of mitochondrial complexes can lead to reduced ATP production and an increase in the production of free radicals [6–8]. This excessive ROS production can contribute to oxidative stress and cellular damage, further aggravating the disease. As a result, targeting mitochondria and exploring ways to preserve their functions and reduce ROS production offer promising therapeutic opportunities to address PD and potentially slow down its progression.

The MitoPark (MP) mouse is a genetic PD model in which mitochondrial function is disrupted in dopaminergic neurons by the elimination of the nuclear genome-encoded mitochondrial transcription factor A (Tfam) gene [9] specifically in midbrain dopaminergic neurons. The Tfam gene is encoded in the nuclear genome, produces a protein that is imported into mitochondria, and acts as a DNA binding protein essential for both transcription and maintenance of mitochondrial DNA (mtDNA) [10]. Disruption of the Tfam gene leads to decreased mitochondrial transcription and subsequent respiratory chain failure [11, 12]. Interestingly, MP mice appear normal at birth, with no increased embryonic or neonatal lethality and they do not exhibit PD-like symptoms at weaning or as young adults. However, at 12 weeks of age, compared to wild type mice, MP mice show significantly decreased locomotion and rearing behavior when evaluated in activity cages. As they reach 20 weeks, more evident phenotypic manifestations such as tremors, twitching, and abnormal gait start to become apparent [13, 14]. MP mice thus successfully mimic the progression of PD in humans, with adult-onset and slow progression of neurological symptoms [9]. This mouse model allows for studies of the role of dopaminergic neuron degeneration, as well as investigations into dopaminergic neuron pathology and its behavioral effects over an extended time course [15–17].

Glucagon-like peptide-1 receptor (GLP-1R) agonists have effectively treated type 2 diabetes mellitus (T2DM) for many years. More recently, growing evidence has supported the potential of GLP-1R agonists as a new treatment strategy for PD [18–20]. Such emerging research suggests that GLP-1R agonists may hold promise in addressing the underlying mechanisms and symptoms associated with PD. Whereas most of the studies suggest that GLP-1R agonists potentially affect PD progression by reducing neuroinflammation, other investigations emphasize the critical role of mitochondrial functions in the disease [21–23]. However, the precise mechanisms that underlie the action of GLP-1 related drugs on mitochondria remain unclear.

In prior investigations, it was observed that the subcutaneous administration of Exenatide results in cerebrospinal fluid (CSF) levels of approximately 2−3% of concurrent plasma levels in humans [24] and rats [25]. These concentrations are most effectively attained through steady-state, long-term drug administration. In the context of this, the administration of Exenatide once every two weeks, utilizing its sustained-release formulation PT320, promptly reached steady-state levels in plasma [26–28]. Notably, this approach demonstrated significantly higher concentrations in the central nervous system (CNS) compared to the twice-daily administration of immediate-release Exenatide, which is clinically used as Byetta [25].

In this communication, we further document changes in mitochondrial function and structure, and efficacy of GLP-1R agonists by evaluating the clinically approved drug Exenatide in its two-week sustained release PT320 formulation. We found that PT320 treatment delays the reduction of tyrosine hydroxylase (TH) expression, decreases ROS levels, and prevents mitochondrial cytochrome c release in the dopaminergic denervation of the striatum in MP mice. Although PT320 did not affect the reduction in mitochondria numbers, it significantly alleviated mitochondrial dysfunction and preserved mitochondrial morphology during the process of progressive dopaminergic denervation in MP mice. Genetic analysis further revealed that PT320 administration to 8-week-old MitoPark mice could regulate the expression of optic atrophy type 1 (Opa1) and mitochondrial fission 1 (Fis1) genes, both of which are responsible for mitochondrial morphology. Specifically, Opa1 not only plays a role in maintaining mitochondrial homeostasis but also inhibits cytochrome c release through cristae remodeling [29]. These findings reinforce the potential of PT320 as a novel therapeutic approach to preserve mitochondrial function and morphology, thereby mitigating the reduction of dopaminergic denervation in the disease progression of PD.

## Methods

### Animals

The breeding process for generating MP mice has been previously described [9, 15, 16]. The generation of MP mice with specific deletion of the Tfam gene in dopaminergic neurons expressing dopamine transporter (DAT) was achieved through a crossbreeding process between mice carrying the DAT promoter-driven Cre recombinase and mice with the floxed Tfam gene. This selective deletion of the Tfam gene within dopaminergic neurons expressing DAT has a specific and significant impact on mitochondrial function. Breeding pairs to generate MP mice were provided to the National Defense Medical Center (NDMC) in Taiwan from a colony maintained within the Intramural Research Program of the National Institute on Drug Abuse, National Institutes of Health (NIDA, NIH, Baltimore, MD, USA). Age-matched wild-type mice were used as controls in these experiments. All animal protocols were approved by the NDMC Animal Care and Use Committee (IACUC 20-128 and IACUC 22-008) in accordance with NIH guidelines (DHEW publications 85-23) Animals were housed in our AAALAC accredited animal facility at NDMC. For stage divisions and times at which cellular and molecular studies were performed, as well as times when PT320 was administered, see Supplementary Fig. 1A and B.

Based on our previous studies [30], clinical grade PT320 was administered subcutaneously once every 2 weeks. The amount of Exenatide in PT320 was 0.6 mg/kg, which accounts for only 2% of the drug available within the PT320 formulation. The remaining 98% are polymers within PT320 that control the release rate of Exenatide. Consequently, the total administered dose biweekly was 30 mg/kg of PT320. Our prior studies have demonstrated that this drug dose (0.6 mg/kg of Exenatide within 30 mg/kg PT320) maintains steady-state levels [24–26]. It is important to note that PT320 microsphere powder does not dissolve in aqueous solution, necessitating the preparation of a fresh PT320 formulation for each dosing and the immediate vortexing before each animal injection.

### Western blots

Mice were euthanized via decapitation, and their tissue was rapidly frozen using liquid nitrogen immersion. The striatal tissue was lysed using RIPA buffer (TAAR-ZBZ5, Biotools Co., Ltd, Taipei, Taiwan) containing a protease inhibitor cocktail (ab20111, Abcam). Protein concentration was quantified using the BCA protein assay kit (K813, Biovision, Milpitas, CA, USA). Subsequently, 30 µg of proteins were mixed with 2X Laemmli sample buffer (1610737, Bio-Rad, Hercules, CA, USA) and denatured at 95°C for 5 minutes. The proteins were then subjected to electrophoresis on a 10% SDS-polyacrylamide gel and transferred onto a polyvinyl difluoride (PVDF) membrane.

Following a 1-hour incubation at room temperature (RT) with blocking buffer (3% milk in tris-buffered saline with Tween 20), the membranes were exposed to primary antibodies against TH (1:1000, rabbit, ab75875, Abcam, Cambridge, UK) and β-actin (1:5000, rabbit, ab8227, Abcam) overnight at 4°C. After three washes with tris-buffered saline with Tween 20 (TBST), the membranes were incubated with HRP-linked anti-rabbit IgG antibodies (1:20000, ab6721, Abcam) for 1 hour at RT. Subsequently, the membranes were developed using Trident femto Western HRP Substrate (GTX14689, Genetex, Hsinchu, Taiwan) and imaged using UVP ChemStudio Plus (Analytik Jena, Jena, Germany). The obtained results were normalized to β-actin levels, which served as a loading control.

### Immunofluorescence (IF) assay

Mice were subjected to cardiac perfusion with 50 mL PBS and 50 mL of 4% paraformaldehyde (PFA) in PBS. Brain tissues were collected and fixed again with 4% PFA in PBS overnight. After dehydrating with 10%, 20%, and 30% sucrose in PBS, the brains were embedded in optimal cutting temperature (OCT) compound (4583, Sakula Finetek, Torrance, CA, USA) for frozen slices. After permeabilization with 0.2% Triton X-100 in PBS for 15 mins at room temperature (RT), brain sections were then incubated with 3% goat serum (ab7481, Abcam) and 3% BSA in 0.2% Triton X-100 in PBS for blocking. Sections were labeled with primary antibodies including TH (1:500, rabbit, ab137869, Abcam), ATPB (1:200, mouse, ab14730, Abcam), DARPP-32 (1:500, rabbit, GTX133350, Genetex), and Ctip2 (1:200, rat, ab18465, Abcam) at 4°C overnight, followed by incubation with fluorescent dye-conjugated secondary antibodies for 2 h at 37°C. Finally, the slices were mounted with Fluoroshield™, which contains the nuclear dye 4′,6-diamidino-2-phenylindole (DAPI) (GTX30920; Genetex) for fluorescence detection. As a negative control, the primary antibody was omitted and observers were blinded. All the fluorescence was imaged in the sections using the THUNDER Imaging Systems (Dmi8 S, Leica, Wetzlar, Germany) by an observer who was blinded to all three groups.

### ROS measurements

ROS measurements were performed immediately after the preparation of brain slices. Mice were sacrificed and the brains were rapidly and gently removed. Coronal 280 µm slices were cut (VT 100, Leica, Wetzlar, Germany) in a chamber filled with cold cutting solution. (in mM: sucrose 194, NaCl 30, KCl 4.5, MgCl_2_ 1, NaH_2_PO_4_ 1.2, glucose 10, and NaHCO_3_ 26). The slices were then transferred to a chamber with oxygenated artificial cerebrospinal fluid (aCSF) solution (aCSF; in mM: NaCl 126, KCl 3, MgCl_2_ 1.5, CaCl_2_ 2.4, NaH_2_PO_4_ 1.2, glucose 11, NaHCO_3_ 26) at 30 °C.

Slices were incubated with the ROS indicator CM-H2DCFDA (‘5- (and-6) -chloromethyl-2’, 7’ dichlorodihydro fluorescein diacetate [DCFDA], acetyl ester, Molecular Probes, Invitrogen, Waltham, MA, USA) 5 µM for 30 min in the dark. The measurements were carried out in a fluorimeter (SYNERGY HT, BioTek, Winooski, VT, USA). The wavelengths for excitation and emission used in the DCFDA fluorescence assay were Ex/Em: 485/ 505 nm, respectively.

### Transmission Electronic Microscopy Image (TEM)

Brains were removed from MP and age-matched wild-type mice at 8, 12, and 18 weeks old. The dorsal striatal region was isolated and cut into ~1mm^3^ cubes. Tissues were fixed in 2% paraformaldehyde (PFA) and 2.5% glutaraldehyde in PBS and thoroughly mixed for TEM analysis. Fixed samples were washed in 0.1 M sodium cacodylate and then post-fixed with 2% osmium tetroxide. After dehydration, the samples were filtered in a graded series of Epon resin (EMS, Hsin An Instruments Co., Ltd, Taiwan) for 2 days, and finally embedded in fresh Epon resin and polymerized at 60 °C for 48 h. Purified tissues were re-suspended in 2% PFA and sectioned at 80 nm thickness to allow attachment to a copper grid (EMS, Hsin An Instruments Co., Ltd, Taiwan). Finally, the sections were examined with a Hitachi HT7700 transmission electron microscope (Hitachi, Tokyo, Japan) at the Pathology Department of Tri-Service General Hospital.

### Morphometric analysis

Morphometric analyses of TEM images were conducted using NIH Image J Software on a sample of 10-15 systematically, uniformly, and randomly selected images. Mitochondrial density was assessed by quantifying the number of mitochondria in the area of a TEM image. Furthermore, the mitochondrial types were identified using the point counting method. The classification of mitochondria into four types is based on the completeness of the mitochondrial outer membrane and the thickness of the cristae [31, 32]. These conditions are closely linked to the mitochondrial structure and can provide insights into mitochondrial function.

### Preparation of brain tissue for respirometry studies

Tissue was prepared as described previously [33]. Mice were anesthetized and the striatal region was extracted. The tissue was cut into small pieces and weighed. MIR buffer was added (EGTA 0.5 mM, MgCl_2_. 6H_2_O 3 mM, Lactobionic acid 60 mM, Taurine 20 mM, KH_2_PO_4_ 10 mM, HEPES 20 mM, d-sucrose 110 mM, BSA, fatty acid free 1g/ L) to achieve a 1 mg per 10 µl concentration. Using a homogenization procedure in microcentrifuge tubes, with three gentle strokes with a syringe equipped with a 25G needle to achieve complete homogenization, 6 mg of tissue (60 µl of homogenate) were used for the following studies.

### High resolution respirometry

The measurement of oxygen consumption was conducted at 2-second intervals using Oroboros respiratory analyzers (Oroboros Instruments, Innsbruk, Austria) equipped with polarographic oxygen sensors. The results were expressed as mass-specific oxygen flux (pmol s^−1^ mg^−1^). The assays were initiated by injecting oxygen into each chamber, effectively elevating the oxygen concentration to >500 nmol ml^−1^ before a substrate-uncoupler-inhibitor titration (SUIT) protocol. To ensure that oxygen availability wasn't limited by diffusion, the chambers were re-oxygenated, maintaining the oxygen concentration within the range of 100–500 nmol ml^−1^ [34]. The analyzer was calibrated daily in an air-saturated solution before experimentation, and each assay was performed at 37°C, with chamber stirrers set at 500 rpm.

### Oxygen consumption rate (OCR) measurement

N27, a dopaminergic line of cells, were seeded into specialized 8-well plates at a density of 10^4^ cells/well, pretreated with or without 50 nM Exendin-4 (Ex-4), and allowed to adhere overnight. After attachment, cells were treated with or without 30 nM 6-hydroxydopamine (6-OHDA) for an additional 3 h. Oxygen consumption was determined using a Seahorse XF HS Mini Analyzer (Agilent Technologies, Savage, MD, USA) as previously described [35]. Prior to OCR measurement, culture medium was changed to serum-free RPMI-1640 containing 1 mM sodium pyruvate, without glucose and sodium bicarbonate. Plates were then incubated for 1 h at 37 °C in a CO_2_-free atmosphere. The OCR profiles in response to injection of oligomycin (1 µM), carbonyl cyanide-4-(trifluoromethoxy) phenylhydrazone (FCCP) (0.5 µM), and a combination of antimycin A (1 µM) and rotenone (1 µM) were evaluated. OCR was measured using four 2 min cycles of mix and measurements following each injection.

### RNA preparation

The striatal tissue was homogenized using a Bullet Blender® BBX24 (Next Advance, Inc., Raymerton, NY) with 0.5 mL of TRIzol™ Reagent (15596026, Thermo Fisher Scientific, Waltham, MA, USA). RNA extraction followed a previously described method [36]. The resulting pellets were resuspended in 10 μL of nuclease-free water. The concentration and purity of the RNA samples were assessed using the NanoDrop 2000 (Thermo Fisher Scientific). Finally, the samples were stored at -80°C until further processing.

### Next-Generation Sequencing (NGS)

Before the analysis, RNA degradation and integrity were monitored by Qsep 1 RNA Cartridge (R1 Cartridge, Taipei, Taiwan). RNA sequencing was performed using the Illumina Stranded mRNA Prep (20040534; Illumina, San Diego, CA, USA) following the manufacturer’s recommendations, and index codes were generating with attributing sequences for each sample. The original data from high-throughput sequencing (Illumina NovaSeq 6000 System) was transformed into raw sequenced reads by the CASAVA base and stored in FASTQ format [37, 38]. These data have been submitted to the NCBI BioProject database. The NCBI SRA accession number for the data is PRJNA1007895. After cleaning up low-quality reads and eliminating poor-quality bases [39, 40], the obtained high-quality data were used for subsequent analysis.

### Quantitative Real-Time Polymerase Chain Reaction (qPCR)

The TaqMan™ MicroRNA Reverse Transcription Kit (4366596, Thermo Fisher Scientific) was used to generate cDNA from the striatum of 8-week-old mice in each group. qPCR was performed using 1 µg cDNA, WizPure™ qPCR Master (SYBR) (W1711, Wizbiosolutions, Gyeonggi-do, Republic of Korea), and specific primers (Supplementary Table 1) were used for mitochondrial complex I-V and for mitochondrial morphology control genes to assay expression. The ddCT method [41–43] was used to determine relative gene level differences of each group.

### Statistics

All the tests were analyzed using a one or two-way analysis of variance (ANOVA) followed by a Bonferroni post hoc test for multiple comparisons and performed using appropriate software (GraphPad Prism 6.01, GraphPad Scientific). A *p*-value <0.05 was considered statistically significant.

## Results

### PT320 delayed the loss of TH expression in striatum and substantia nigra pars compacta (SNc)

In accord with our previous studies [9, 14, 15, 30, 44], we referred to the disease course in PD patients to divide the PD-like progression in MP mice into three stages based on their PD-like symptoms. The stage divisions are shown in Supplementary Fig. 1A. The early stage corresponds to the preclinical stage where no obvious phenotypic characteristics are observed. In the middle stage, protein expression and neural functions begin to decline, and the mice start to exhibit small motor defects. The late stage represents the motor phenotype stage, characterized by severe defects.

**Fig. 1:**
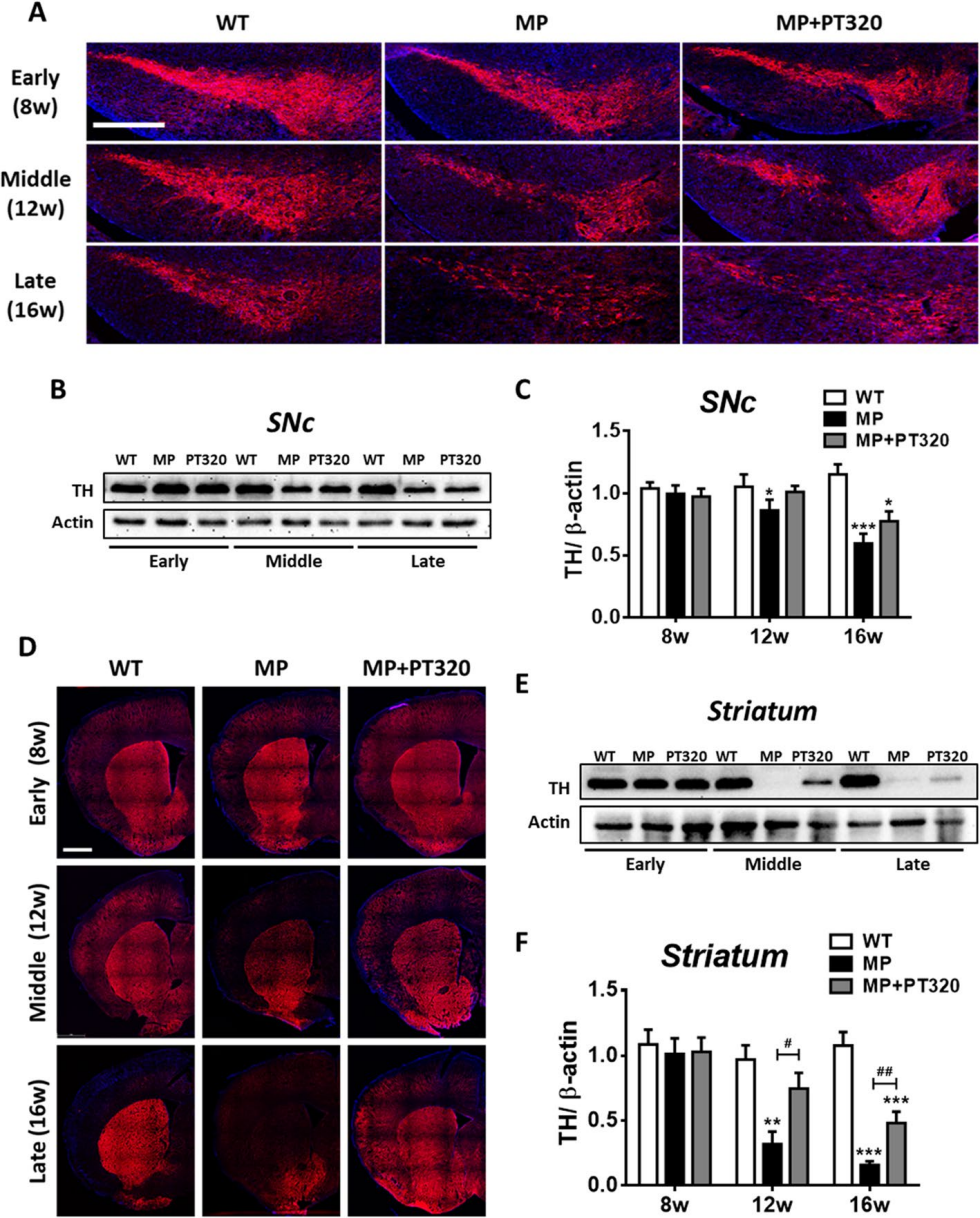
Changes in TH protein expression at three MitoPark and PT320-treated MP mouse PD stages. Results show at the level of the substantia nigra pars compacta (SNc), TH starts to decline during the middle stage. The PT320 treatment showed significant differences at the late stage. The TH expression of the striatum area showed little change at the early stage but substantial changes with PT320-treated groups in the middle and late stages, as compared to untreated MitoPark animals. **A** Representative immunofluorescent images of TH expression in the SNc. Scale bar: 0.5 mm (**B**) Western blot results of TH expression and **C** The statistical results in SNc. **D** Representative immunofluorescent images of TH expression in the striatum. Scale bar: 1 mm (**E**) Western blot results of TH expression and **F** The statistical results in striatum. One-way analysis of variance (ANOVA) followed by Bonferroni post hoc test for multiple comparisons. WT vs MP or MP+PT320: *, *p* < 0.05; **, *p* < 0.005; ***, *p* < 0.001; MP vs MP+PT320: #, *p* < 0.05; ##, *p* < 0.005. (*N* = 3-5)

The timeline of PT320 treatment and the following experimental protocols are shown in Supplementary Fig. 1B. In our previous study [30], PT320 was administered to ameliorate abnormal behaviors in MP mice. To assess the protective effects of PT320, histological analysis was again performed to determine whether dopaminergic neurons in the striatum and SNc could be preserved. As shown in Fig. 1A, there was no evident difference in TH-positive neuron expression among all the groups at the early PD stage in the SNc. However, TH started to decline in the middle stage in both the MP and MP + PT320 groups. Quantitative protein expression results are presented in Fig. 1B and C. Notably, there were no differences across groups at the early PD stage; however, a statistically significant and progressive decline in the MP group was evident during the middle and late stages. In contrast, the MP + PT320 group showed a smaller decline, suggestive of protective effects of PT320 treatment in the SNc at the late disease stage (Fig. 1C). The TH images and protein levels in the striatum are displayed in Fig. 1D, E, and F. TH-positive neuron expression showed earlier changes in the striatum compared to the SNc. PT320 treatment clearly attenuated the loss of TH in the middle and late stages. Together, these data confirm the protective effect of PT320 in MP mice and demonstrate that the protective effects occur earlier in the striatum compared to the SNc.

### PT320 reduced ROS production and inhibited the cytochrome c release in the striatum

**Fig. 2:**
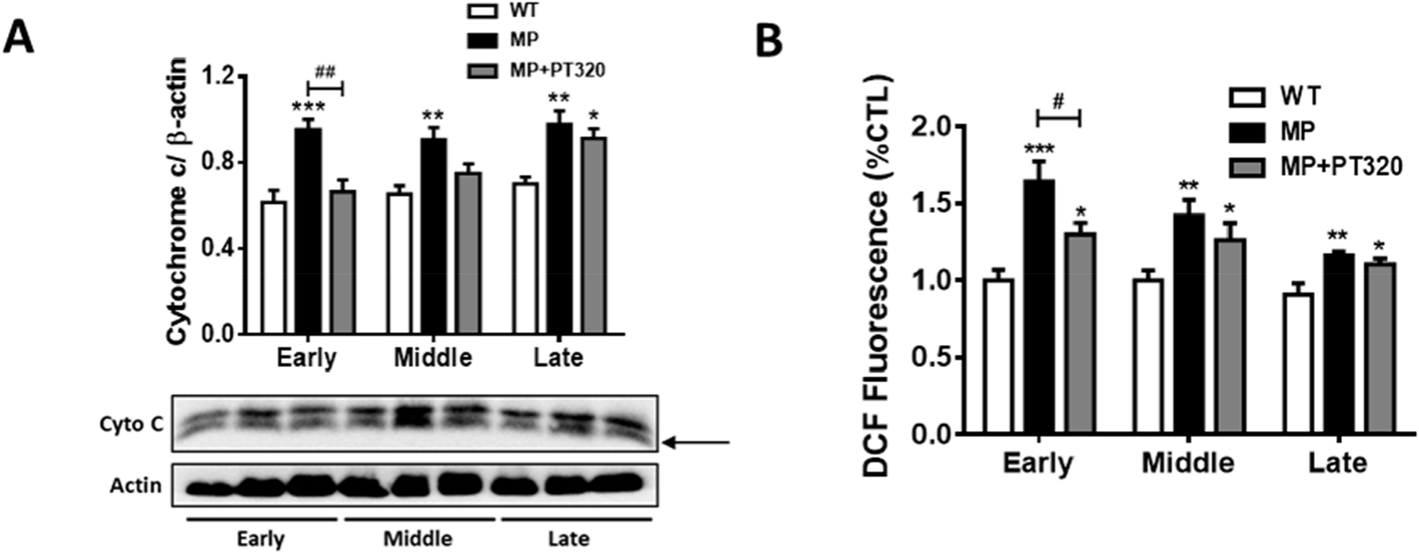
Although TH proteins showed no difference in the early stage of MP mice and the PT320-treated groups, there was a significant difference in cytochrome c release and ROS production at 8 weeks of age, indicating a potential molecular change during the early stage of disease progression. **A** Cytochrome c protein levels in different age groups of MitoPark and PT320-treated mice. (12kDa, the lower bands) (**B**) Measurement of ROS production through DCFDA fluorescence. One-way analysis of variance (ANOVA) followed by Bonferroni post hoc test for multiple comparisons. WT vs MP or MP+PT320: *, *p* < 0.05; **, *p* < 0.005; ***, *p* < 0.001; MP vs MP+PT320: #, *p* < 0.05; ##, *p* < 0.005. (*N* = 5)

The integrity of mitochondrial function is closely related to the integrity of its membrane. Cytochrome c serves as an indicator to assess the integrity of the mitochondrial membrane. Additionally, the release of cytochrome c can act as an ROS scavenger. Both of these indicators are linked to mitochondrial functions in cells. In Fig. 2A and B, our results show that statistically significant differences in both cytochrome c and ROS levels in the striatum were observed between WT and MP mice at the early stage. However, these findings differed from the results obtained for TH. The significant difference between MP and MP + PT320 groups was only observed in the early stage. These results suggest that the protective effect of PT320 occurs primarily in the early stage, and initiating treatment during the middle or late stages may not be as effective as early-stage treatment.

### The mitochondrial number declined in MP mice, while PT320 preserved mitochondrial morphology

**Fig. 3:**
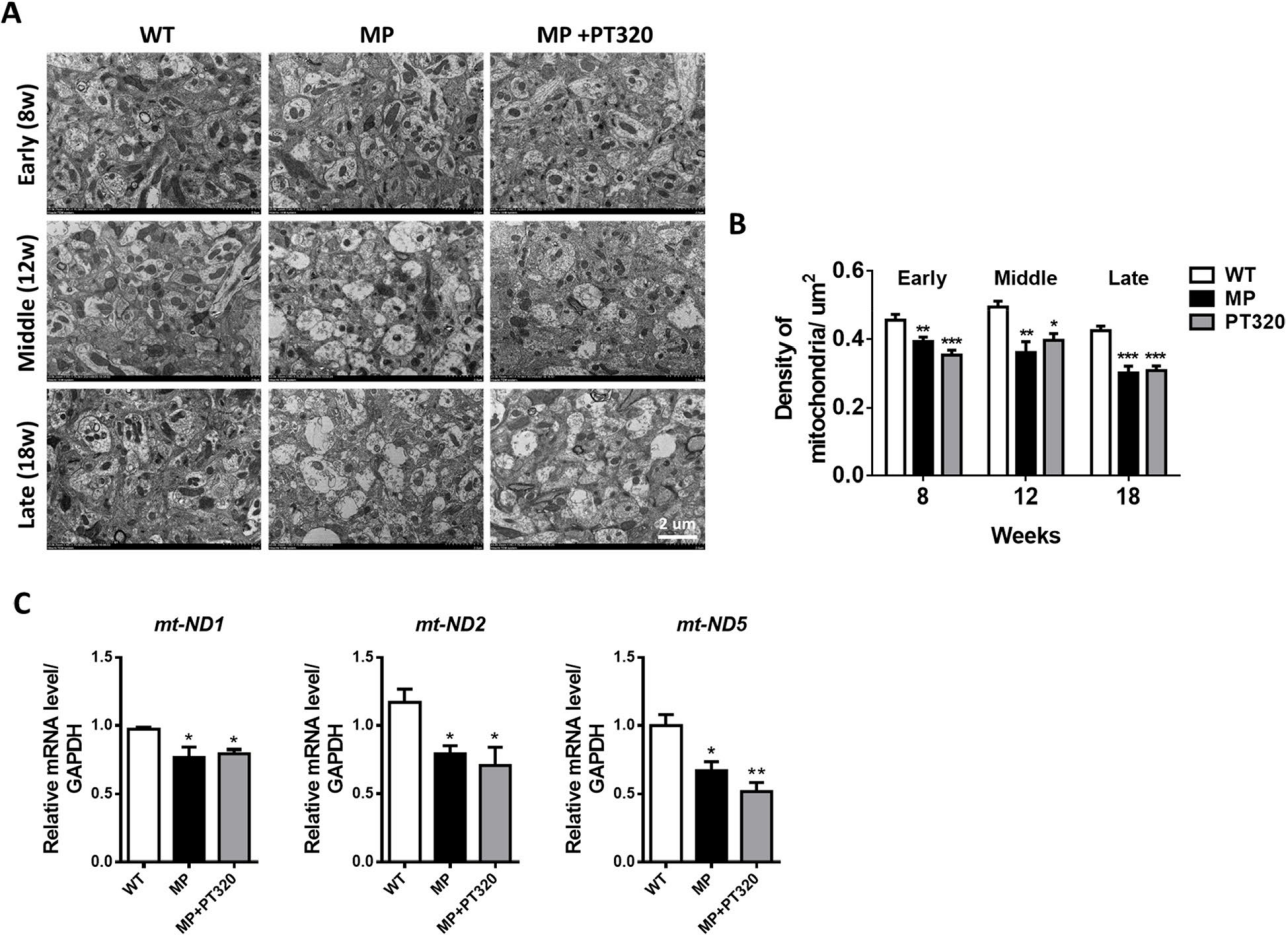
Transmission electron microscopy (TEM) was used to examine the number of mitochondria in the striatum. The results indicate that PT320 treatment does not prevent the decline in mitochondrial numbers. Scale bar: 2 µm (**A**) Representative TEM images are shown for different stages, including wild-type, MitoPark, and PT320-treated MitoPark. **B** Statistical analysis shows the mitochondrial numbers for these different stages. **C** The mitochondrial encoding genes ND1, ND2, ND5 expression in early stage. One-way analysis of variance (ANOVA) followed by Bonferroni post hoc test for multiple comparisons. WT vs MP or MP+PT320: *, *p* < 0.01, **, *p* < 0.05, ***, *p* < 0.001. (*N* = 3)

To further investigate the effects of PT320 on mitochondrial functions, we conducted an examination of mitochondria in the striatum using TEM images. The numbers of mitochondria were quantified, and the results are displayed in Fig. 3A and B. Despite no observable PD characteristics at the early stage in MP mice, there was a significant reduction in the number of mitochondria. PT320 administration did not prevent this decline in mitochondria numbers. The data indicated no significant differences among all stages of MP and MP + PT320 groups (Fig. 3B). To validate the results of TEM analysis, we used qPCR to evaluate the expression of mitochondrial-encoded genes NADH-ubiquinone oxidoreductase chain 1, 2, and 5 (mt-ND1, mt-ND2, and mt-ND5), which can serve as indicators of mitochondrial numbers. Figure 3C demonstrates the qPCR results, showing a statistically significant decrease in the expression of mt-ND1, ND2, and ND5 in MP mice, with no significant difference observed between the MP and MP + PT320 groups.

**Fig. 4:**
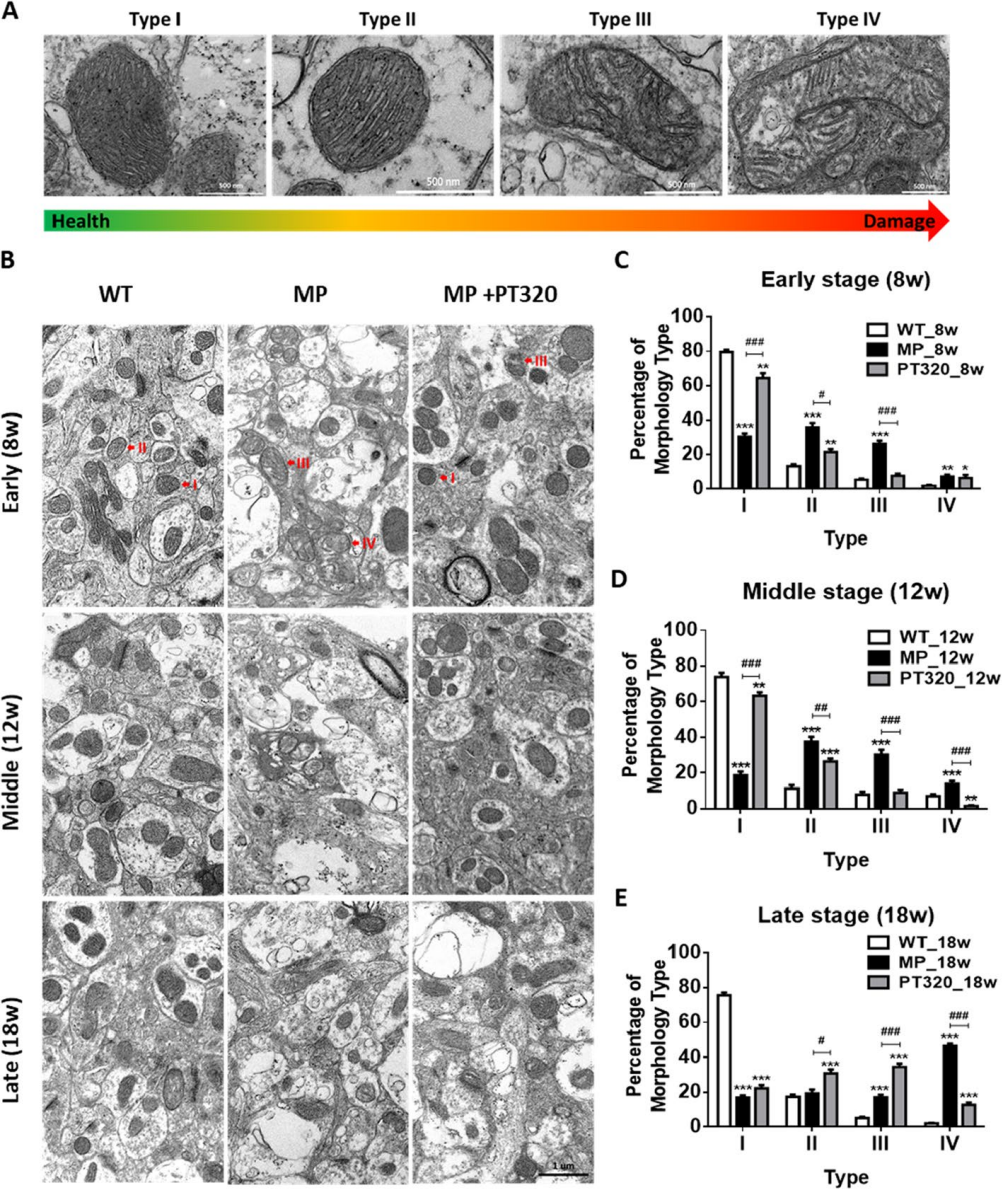
TEM analysis of mitochondrial structural changes in the striatum region in wild-type (WT), MitoPark, and PT320-treated MitoPark mice. The mitochondrial structures can be divided into four types as detailed in Results. Type I is the normal structure of healthy mitochondria. Type IV is the most damaged mitochondria, with disrupted and discontinuous outer membranes and deficient cristae. The red arrows indicate the different types of mitochondria. **A** Representative TEM images of four types of mitochondrial morphologies. Scale bar: 500 nm (**B**) Representative TEM images of different stages of the mitochondria morphologies in three groups. Scale bar: 1 µm (**C**) Percent of mitochondria type in early stage. **D** Percent of mitochondria type in middle stage. **E** Percent of mitochondria type in late stage. One-way analysis of variance (ANOVA) followed by Bonferroni post hoc test for multiple comparisons. WT vs MP or MP+PT320: *, *p* < 0.01; **, *p* < 0.05; ***, *p* < 0.001; MP vs MP+PT320: #, *p* < 0.01; ##, *p* < 0.05; ###, *p* < 0.001. (*N* = 3)

The analysis of TEM images not only focused on mitochondrial numbers but also assessed changes in mitochondrial structure. Figure 4A illustrates the classification of mitochondria into four distinct types (I-IV) based on their structural characteristics. Type I mitochondria exhibit a normal appearance with longitudinally oriented and tightly packed cristae, which are crucial for energy production. Type II mitochondria display abnormal morphology, lacking a distinct shape and having nonuniform sizes. The cristae in Type II mitochondria appear swollen, irregular, and lose their longitudinal orientation. These cristae may also exhibit a loss of tightness and regular spacing. Type III mitochondria show a wide range of shapes and sizes, demonstrating significant variation. They have a discontinuous outer membrane and undergo homogenization and fragmentation of the cristae, the internal membrane structures. The matrix, the innermost compartment of the mitochondria, appears swollen and has an electron dense appearance. Type IV mitochondria have a disrupted outer membrane, leading to compromised structural integrity. They manifest a deficiency in cristae, with a notable reduction or absence of the internal membrane structures.

The results of the characterization of these four types of mitochondria in different stages of the WT, MP, and MP + PT320 groups are depicted in Fig. 4B. Additionally, Fig. 4C, D, and E show the percentage of each mitochondrial type in different stages and groups. In the early stage (Fig. 4C), both WT and MP + PT320 groups show that over seventy percent of mitochondria are type I, whereas the MP mice exhibit less than fifty percent. Furthermore, the MP mice display a corresponding increase in type II and type III mitochondria, indicating disrupted mitochondrial morphology at the early PD stage in MP mice. In the middle stage (Fig. 4D), the MP group exhibits a retention of approximately twenty percent of type I mitochondria. The presence of PT320 continues to demonstrate a “protective” effect on mitochondrial morphology during this stage. However, in the late stage (Fig. 4E) of the MP + PT320 group, there is a substantial decline in the proportion of type I mitochondria. Meanwhile, both type II and type III mitochondria show an increase in abundance. Furthermore, during this late stage, the major type of mitochondria in the MP mice becomes type IV, indicating compromised structural integrity. These observations highlight the progressive deterioration of mitochondrial morphology in MP mice at this stage, even with the presence of PT320, which initially exhibited a protective effect on mitochondrial structure.

### PT320 reduces mitochondrial dysfunction at the early and middle stages

**Fig. 5:**
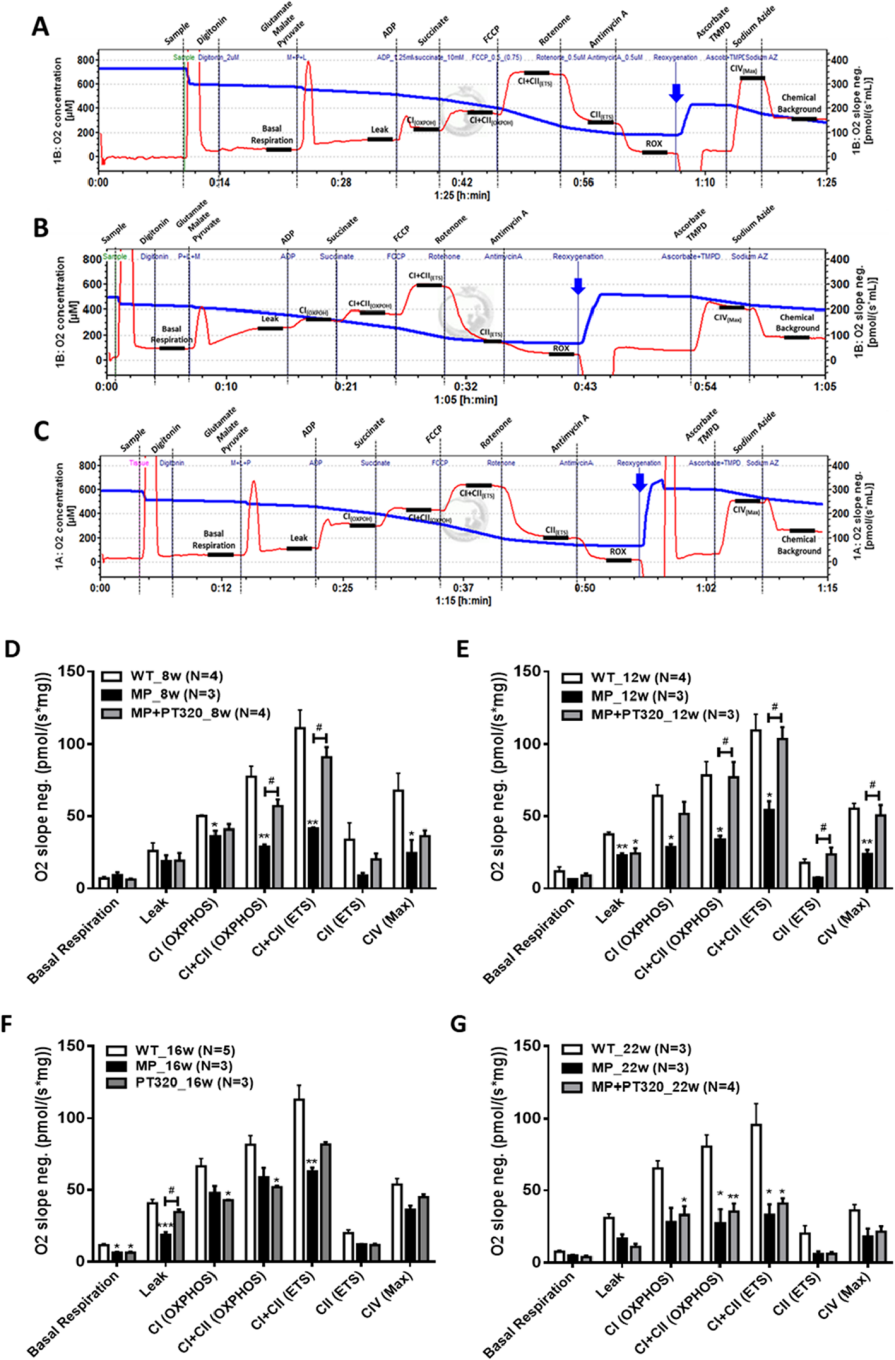
The results of the Oroboros O2k analysis were obtained for striatum tissue samples from wild-type (WT), MitoPark, and PT320-treated MitoPark mice at different ages using the mitochondrial-specific complex protocol. **A** Oroboros O2k representative tracings of 8 weeks old WT mice. **B** O2k representative tracings of 8-week old MP mice. **C** O2k representative tracings of 8 weeks old MP + PT320 mice. **D**-**G** Age-dependent (8w, 12w, 16w and 22w, respectively) mitochondrial-specific O2 flux during basal, mitochondrial complex linked (CI, CII, CIV) and non-mitochondrial respiration. One-way analysis of variance (ANOVA) followed by Bonferroni post hoc test for multiple comparisons. WT vs MP or MP+PT320: *, *p* < 0.01; **, *p* < 0.05; ***, *p* < 0.001; MP vs MP+PT320: #, *p* < 0.01; ##, *p* < 0.05; ###, *p* < 0.001

Next, we performed high-resolution respirometry on striatal tissue. The structure of mitochondria is closely linked to their function, particularly in the context of oxidative phosphorylation (OXPHOS). OXPHOS is a fundamental process that occurs within the mitochondria and is responsible for generating energy in the form of ATP. This tightly coupled process, involving electron transport, proton pumping, and ATP synthesis, ensures that mitochondrial OXPHOS is intimately connected to oxygen consumption. Figure 5A, B, and C display representative tracings of 8-week-old WT, MP, and MP + PT320 mice. The details regarding the concentrations and properties of the drugs used are described in Supplementary Fig. 2.

The quantitative results at different ages and groups are shown in Fig. 5D, E, F, and G. At the early stage of 8 weeks old (Fig. 5D), the mitochondrial-specific O2 flux of MP mice was lower compared to the WT group. MP + PT320 showed a strong protective effect on mitochondrial function, specifically in CI + CII (OXPHOS) and CI + CII (ETS). In the middle stage of 12 weeks of age (Fig. 5E), similar results were observed compared to the early stage, except for significant differences in complex IV between MP and MP + PT320. In the later stages of 16 and 22 weeks of age (Fig. 5F and G), the differences between MP and MP + PT320 started to decline. These results are consistent with the changes in mitochondrial structure noted above, indicating that the protective effects of PT320 may be mediated through early prevention of mitochondrial dysfunctions.

**Fig. 6:**
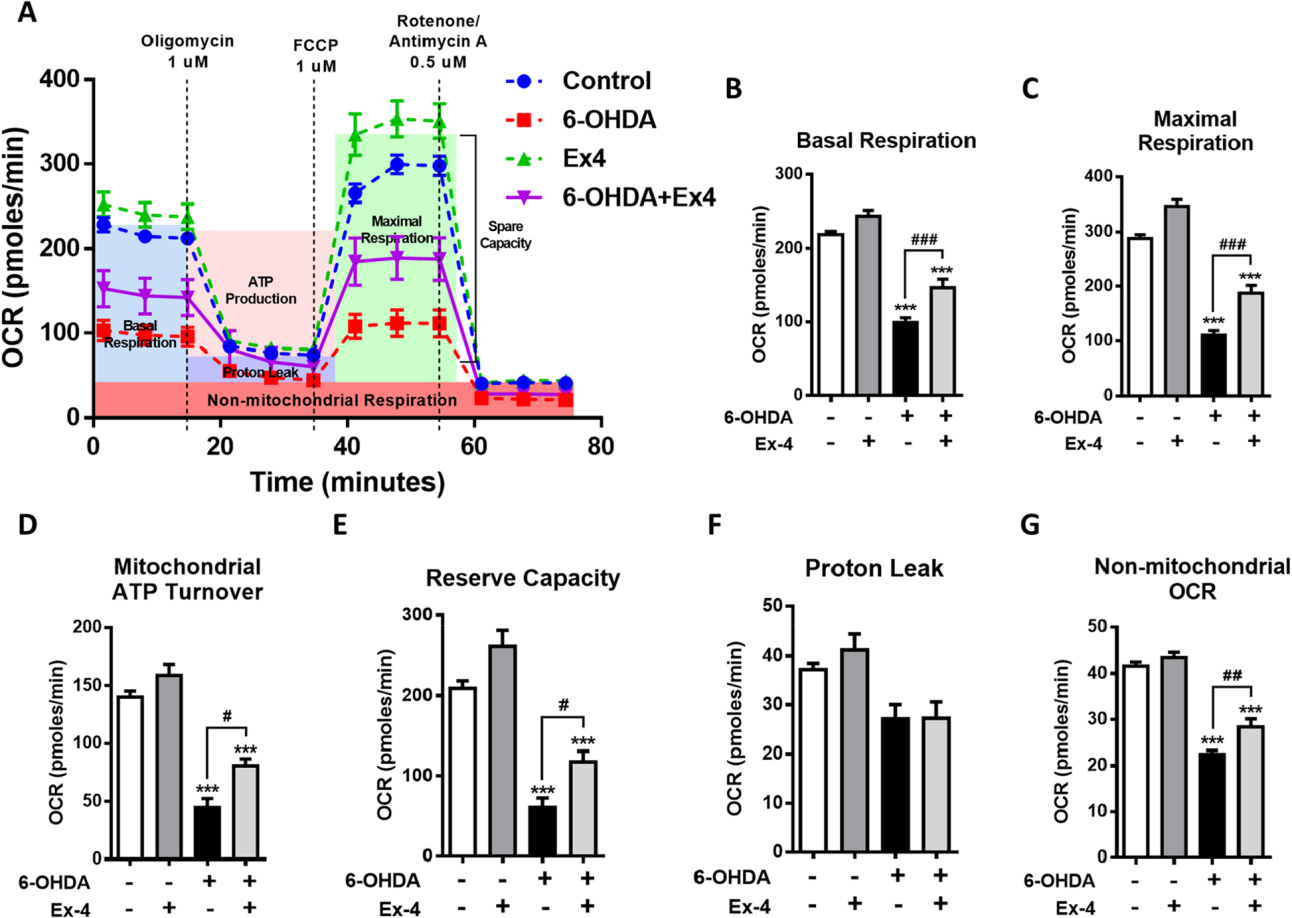
Data indicates that mitochondrial dysfunction occurs as a result of damage induced by 6-OHDA. Importantly, co-treatment with exendin-4 (Ex4) exhibits a protective effect against the loss of mitochondrial function in N27 cells. **A** N27 cells were incubated for 18 hours with or without 30 µM of 6-OHDA and/or 50 nM Ex4. Subsequently, extracellular flux analysis was performed. Oxygen consumption rate (OCR) was measured sequentially after the injection of 25 mM glucose and a set of mitochondrial inhibitors (1 µM oligomycin, 1 µM FCCP, and 0.5 µM each of rotenone and antimycin A) to generate a mitochondrial stress profile. (B–G) Mitochondrial parameters were calculated from the mitochondrial stress profile using the methods described in this study. The parameters assessed were: **B** basal respiration, **C** maximal respiration, **D** mitochondrial ATP turnover, **E** reserve capacity, **F** proton leak and **G** non-mitochondrial OCR. One-way analysis of variance (ANOVA) followed by Bonferroni post hoc test for multiple comparisons. Control vs Control + Ex-4 or 6-OHDA or 6-OHDA + Ex-4: *, *p* < 0.01; **, *p* < 0.05; ***, *p* < 0.001; 6-OHDA vs 6-OHDA+ Ex-4: #, *p* < 0.01; ##, *p* < 0.05; ###, *p* < 0.001. (*N* = 6)

To further confirm the effects of PT320 on dopaminergic neurons. The rat dopaminergic neural cell line N27 was utilized to assess the effects of Ex-4 (non-clinical grade Exenatide) on mitochondrial function at the cellular level. For this in vitro evaluation, 6-OHDA was used as a DA neurotoxic challenge. Figure 6A illustrates the mitochondrial oxygen consumption rate (OCR) under various conditions. The curves of the groups treated with 6-OHDA exhibited a significant decrease. Importantly, the group co-treated with Ex-4 showed a reduction in the rate of decline. Ex-4 not only prevented the decline of both basal and maximal respiration (Fig. 6B and C) but also preserved the ATP turnover rate and reserve capacity (Fig. 6D and E). These results indicate that Ex-4 supports mitochondrial functions, maintains the ATP turnover rate, and reduces the susceptibility to cellular damage caused by 6-OHDA.

### PT320 affects the complex II component proteins rather than regulating mitochondrial complex relative genes

**Fig. 7:**
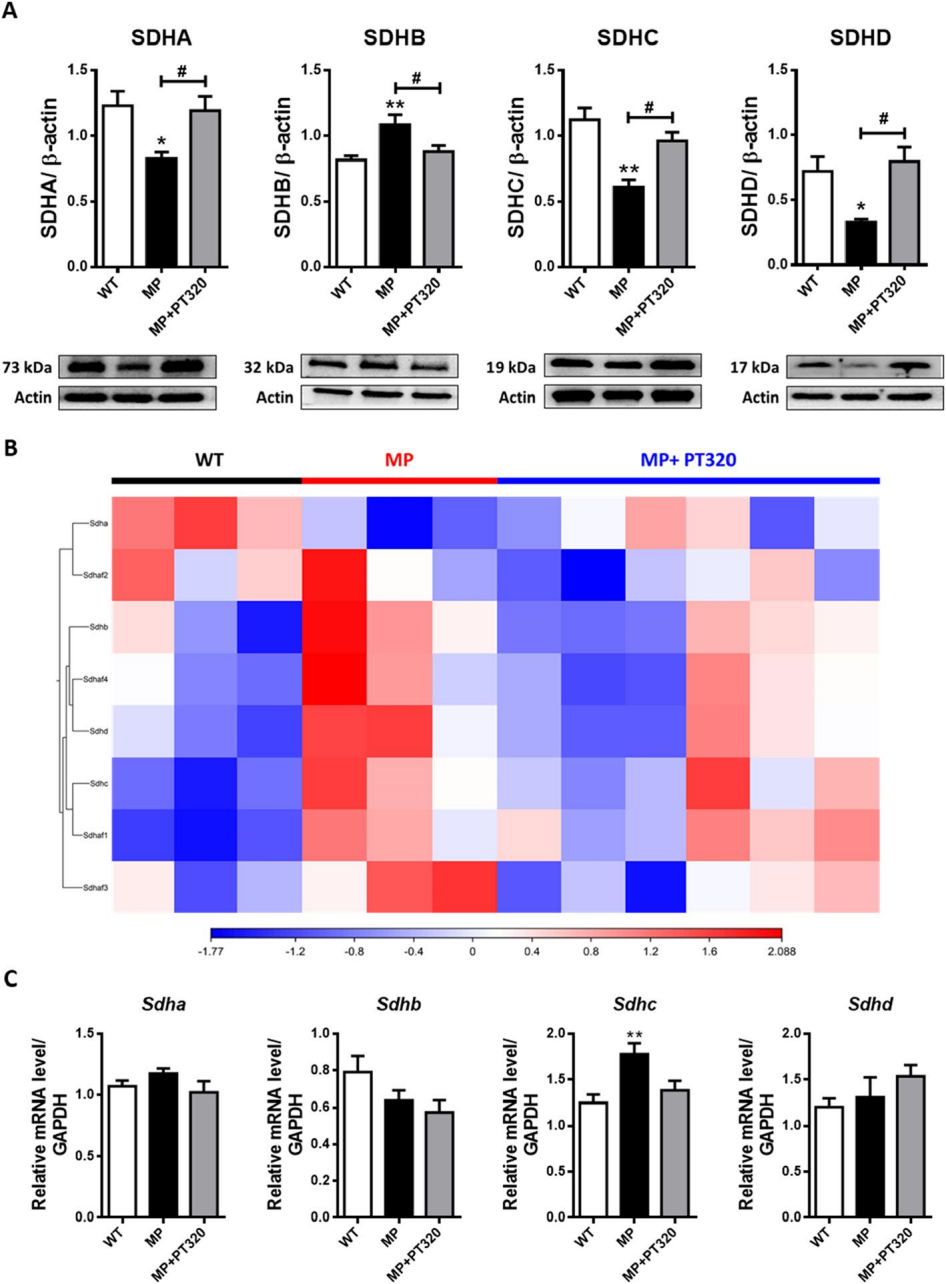
The mitochondrial complex II is composed of Succinate dehydrogenase complex subunits A, B, C, and D (SDHA, SDHB, SDHC, and SDHD). The data here shows inconsistencies between the protein levels and RNA levels, indicating that the protective effects of PT320 may not directly affect the mitochondrial complex. **A** Protein level results of SDHA, SDHB, SDHC, and SDHD. **B** The heat map of the complex II associated RNA expression in Next-Generation Sequencing (NGS) data. **C** The real-time polymerase chain reaction (rtPCR) results of complex II composed genes. (*N* = 3-6)

Based on the mitochondrial functional tests, mitochondria complex II seems to have the earliest response to PT320 administration (Fig. 6B). To further understand how PT320 affects mitochondrial functions, we investigated the expression of mitochondrial complex II component proteins in the early stage of 8 week old mice. The mitochondrial complex II is composed of succinate dehydrogenase complex subunits A, B, C, and D (SDHA, SDHB, SDHC, and SDHD), which are encoded by nuclear DNA (nDNA). As shown in Fig. 7A, the four proteins exhibited significant differences between the MP and MP + PT320 groups. However, the NGS data demonstrated different results. The principal component analysis (PCA) of the indicated samples is shown in Supplementary Fig. 3. Although the MP group was separate from the control (WT) and PT320 (MP + PT320) groups, the differences in the heatmap of mitochondrial complex II-associated RNA were not as obvious (Fig. 7B). To further confirm the results of NGS, we used qPCR to evaluate the expression of the four major genes of the complex II components, *Sdha*, *Sdhb*, *Sdhc*, and *Sdhd*. Figure 7C shows the qPCR results, which are similar to the NGS data. The complex I, IV, and V-related genes were also examined, and the data is shown in Supplementary Fig. 4A, B, and C. These results indicate that although PT320 preserves mitochondrial functions, it might not do so directly through the regulation of complex-related genes.

### PT320 preserves the mitochondrial functions in part by regulating the mitochondrial morphology controller genes Opa1 and Fis1

**Fig. 8:**
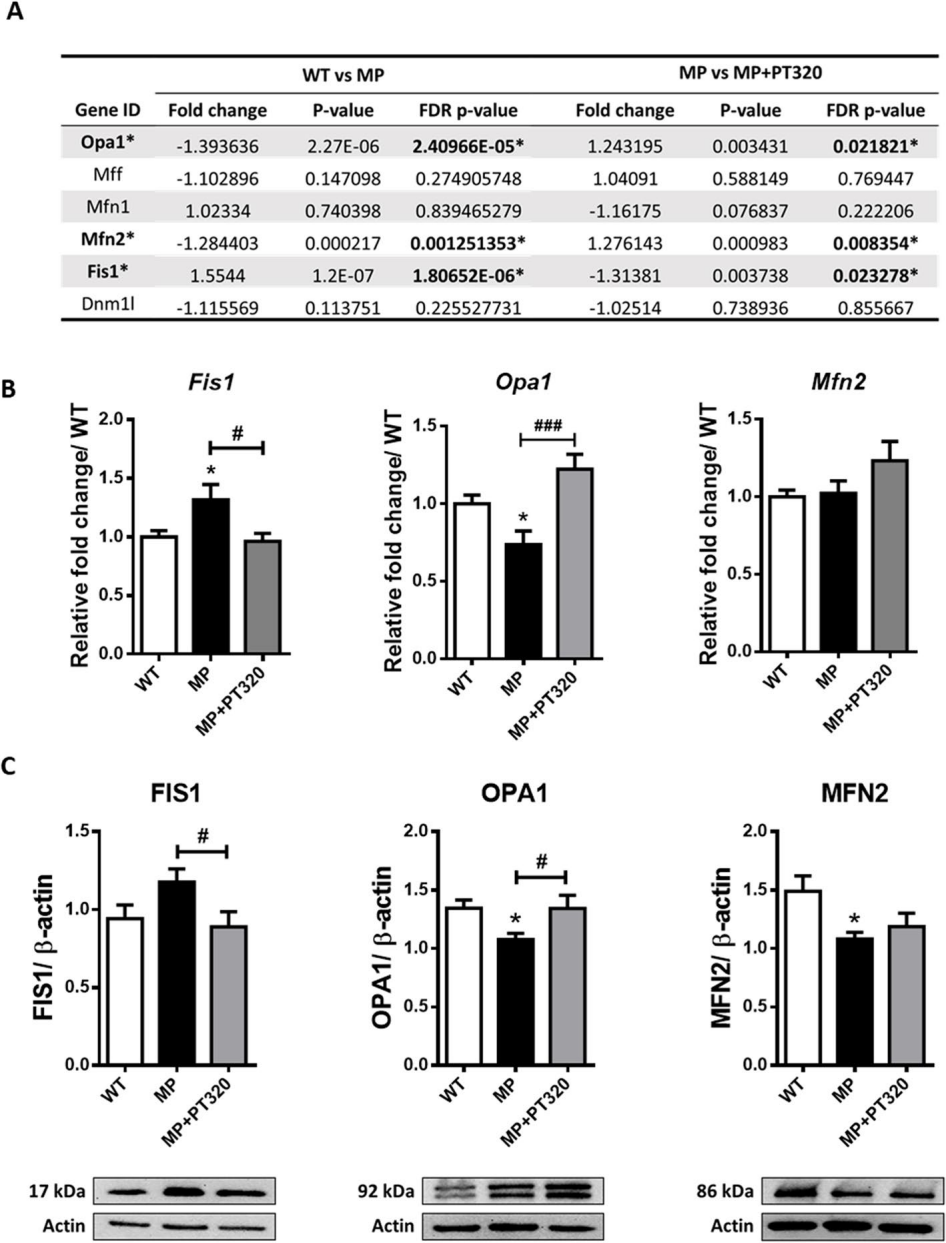
Results of 6 selected genes which are mitochondrial morphology controllers. Although the fold change was not significant, we choose the genes which the FDR *p*-value <0.05 to conduct qPCR analysis, indicating a potential involvement in mitochondrial morphology control. Confirmation of the RNA levels and protein expressions of Opa1, Mfn2, and Fis1, with FDR *p*-values <0.05. The results indicate that PT320 might have a major influence on Opa1 and Fis1, but not on Mfn2. **A** Summary of NGS results of selected genes. **B** The qPCR results of the Opa1, Mfn2, and Fis1 genes. **C** Protein expression of Opa1, Mfn2, and Fis1. (*N* = 3-6)

Other genes that might affect mitochondrial functions are the morphology control genes. These genes not only regulate mitochondrial size and shape but also contribute to mitochondrial homeostasis in cells. Supplementary Fig. 5 shows the heatmap of mitochondrial morphology control genes. The differences in gene expression in the three groups are more evident than the complex-related genes described above. The detailed comparison values of these genes in NGS are shown in Fig. 8A. As seen in Fig. 8A, to perform qPCR, we selected the genes with an FDR *p*-value of less than 0.05, indicating significant differences. The results are shown in Fig. 8B. Among the selected genes, *Opa1* and *Mfn2* are involved in controlling mitochondrial fusion, while *Fis1* is responsible for mediating mitochondrial fission. In our data, the *Fis1* gene was upregulated in MP mice, but PT320 inhibited this gene expression upregulation in MP mice at the early stage. In contrast, the *Opa1* gene was low in expression in MP mice, and PT320 administration upregulated *Opa1* expression, with no significant changes in *Mfn2*. Protein expression was confirmed in Fig. 8C, which was similar to the RNA results, except for MFN2 protein, which showed significantly low expression in both MP and MP + PT320 mice. The in vitro data of N27 cells were also confirmed by 6-OHDA damage and Ex4 treatment (Supplementary Fig. 6). The Ex4 treated groups exhibited similar trends compared to the in vivo data. Taken together, we postulate that PT320 administration preserves mitochondrial functions not directly on mitochondrial complex but by influencing the mitochondrial morphology control genes *Fis1* and *Opa1*.

## Discussion

Mitochondrial dysfunction and energy failure have been repeatedly implicated as the cause of the death of DA neurons in PD [45–50]. Some toxins used to model DA loss in PD, such as 1-methyl-4-phenyl-1,2,3,6-tetrahydropyridine (MPTP) and rotenone, impair respiratory chain function by inhibiting complex I [46, 51–54]. In further support for a “mitochondrial genetics” hypothesis for PD pathophysiology, There is reported higher levels of mitochondrial DNA deletions in nigral neurons from PD patients [55]. Moreover, both Bender et al. [55] and Kraytsberg et al. [56] reported higher levels of mitochondrial DNA deletions in nigral neurons of aged humans with sharp elevations starting shortly before age 70. This correlates with the known risk factor of age in PD.

Tfam is an essential factor for maintaining and replicating mtDNA. Inactivation of Tfam genes causes a reduction in the copy number of mtDNA in cells, resulting in decreased ATP production. Manipulating Tfam is widely used and is highly valuable for studying mitochondrial activity [57–61]. The knockout of Tfam has a direct impact on mitochondrial functions by causing a rapid reduction in the levels of mtDNA-encoded subunits of the OXPHOS complexes. This disruption in mtDNA-encoded components significantly impairs OXPHOS, contributing to mitochondrial dysfunction. In this study, we demonstrated the protective effects of PT320, especially the effects on mitochondria, using MP mice. Our results indicated that PT320 (clinical grade sustained release Exenatide) administration significantly preserved mitochondrial functions without changing the mitochondrial loss that is seen in MP mice. It has been reported that GLP-1R agonists improve mitochondrial functions in T2DM [62–64]. Moreover, these agonists have gained increasing attention in the area of neurodegenerative disease treatment due to their potential involvement in regulating mitochondrial functions [19, 23, 65–68], as mitochondria not only generate most of the cellular ATP, but are involved in multiple other cellular functions that include Ca^2+^ signaling, differentiation, apoptosis, cell cycle, and cellular growth.

In this regard, mitochondrial morphology and functions are closely related, and controlling mitochondrial morphology will also regulate its functions. The inner membrane (IM) of mitochondria forms characteristic inward folds, known as cristae. These cristae are essential for various mitochondrial functions, including ATP production through oxidative OXPHOS. The shape and structure of cristae can change dynamically, reflecting the adaptability of mitochondria to different cellular needs, which supports the concept that cristae are the actual bioenergetic membranes of the mitochondrion. Cristae structure was proposed to increase the inner membrane's surface area, thus enhancing the capacity of OXPHOS [69]. Furthermore, cristae were hypothesized to serve as a specialized compartment ensuring optimal conditions for ATP production by concentrating proteins involved in OXPHOS and reducing the mean distance between different moieties [70, 71].

It appears there might be a discrepancy between the observed mitochondrial morphology changes and the Oroboros O2k data in our studies. Whereas mitochondrial morphology demonstrated differences from the early disease stage to the late stage, the Oroboros O2k data only indicates differences at the early and middle stages. In this regard, it is interesting to note the variations in mitochondrial morphology data based on the four types we classified. The classification depends on cristae density and numbers. In the early and middle stages (Fig. 4C and D), there was a significant increase in the percentage of type I mitochondria in the MP + PT320 group, as compared to the MP group. Meanwhile, type II and type III exhibited decreased percentages of mitochondria in the MP + PT320 group. Type IV mitochondria started to increase during the middle stage of MP mice. However, in the late disease stage (Fig. 4E), the overall differences in mitochondrial morphology remained distinct between the MP and MP + PT320 groups. Moreover, in the MP + PT320 group, the percentage of type I mitochondria dramatically declined, while type IV mitochondria significantly increased. When comparing these findings with the Oroboros O2k data, it is intriguing that the mitochondrial functions in the late disease stage (Fig. 5F and G) showed no differences between MP and MP + PT320 groups. Taking both sets of data into consideration, suggests that type I mitochondria may, indeed, play a central role in energy production, given their pronounced changes in morphology and potential significance.

Mitochondria undergo continuous fission and fusion processes, which collectively form a dynamic equilibrium known as mitochondrial dynamics. This dynamic equilibrium is essential for maintaining mitochondrial homeostasis. The process relies on the intricate interplay of the mitochondrial quality control (MQC) system, composed of various components such as mitochondrial fusion and fission, biogenesis, and mitophagy [72]. The process of MQC acts to eliminate damaged or aging mitochondria and to synthesize new mitochondria, ensuring the stability of mitochondrial quantity, morphology, and internal environment. Mitochondrial fusion leads to the union of the outer and the inner mitochondrial membranes of neighboring mitochondria, and involves two highly conserved dynamin- associated guanosine triphosphatases MFN2 and MFN1 localized to the outer membrane, as well as OPA1 localized to the inner mitochondrial membrane [73]. Fission, leading to mitochondrial division, is regulated by other mitochondrial proteins that include FIS1 [73].

Our study highlights the critical role of mitochondrial morphology control in preserving mitochondrial functions by PT320 in MP mice. *Opa1*, *Mfn2*, and *Fis1* are associated with mitochondrial homeostasis, fine balancing mitochondrial fusion and fission to optimize mitochondrial “health” and functionality; dysregulation is associated with several common genetic mutations in PD. For example, in vitro studies have demonstrated that *Snca* and *Lrrk2*, major genes linked to sporadic PD [74], cause significant mitochondrial fragmentation by enhancing DRP1 function and suppressing OPA1 expression. Likewise, mutations in *Opa1*, usually associated with autosomal dominant optic atrophy, have been linked to altered mitophagy and parkinsonism [75]. Furthermore, aberrant *Mfn2* expression has been reported in metabolic disorders, including type 2 diabetes mellitus and insulin resistance [73], for which Exenatide and GLP-1R agonists are effective in treatments [76]. By either blocking mitochondrial fission or promoting mitochondrial fusion, improvements in mitochondrial morphology, function, and cell viability have been observed [77–79]. In addition to genetic mutations, neurotoxic PD preclinical models induced by substances like rotenone, MPTP, and 6-OHDA also contribute to mitochondrial fission. These models have been shown to cause mitochondrial fragmentation, which can be attenuated by promoting fusion or inhibiting fission processes [80, 81]. Notably, OPA1 plays a multifaceted role within mitochondria. It is not only critical in regulating mitochondrial fusion but also has key functions in mitochondrial Ca^2+^ signaling, mitochondrial membrane potential, oxygen consumption, ATP concentrations, and ROS production by controlling the inner mitochondrial membrane and cristae junctions [82–84]. Together this evidence emphasizes the importance of OPA1 in regulating mitochondrial functions and align with our findings that PT320 influences mitochondrial functions by upregulating OPA1 expression. In this regard, focusing on the regulation of mitochondrial homeostasis represents a potentially effective therapeutic target in PD.

In summary, Exenatide and long-acting GLP-1R agonists have been demonstrated to mitigate PD in both preclinical models and recent human clinical trials via multiple GLP-1R mediated mechanisms [19, 68, 85–87]. By studying the role of GLP-1R agonists in preserving mitochondrial functions, we may contribute to the development of novel treatment strategies that could have a positive impact on PD progression at an early stage.

## Conclusion

**Fig. 9:**
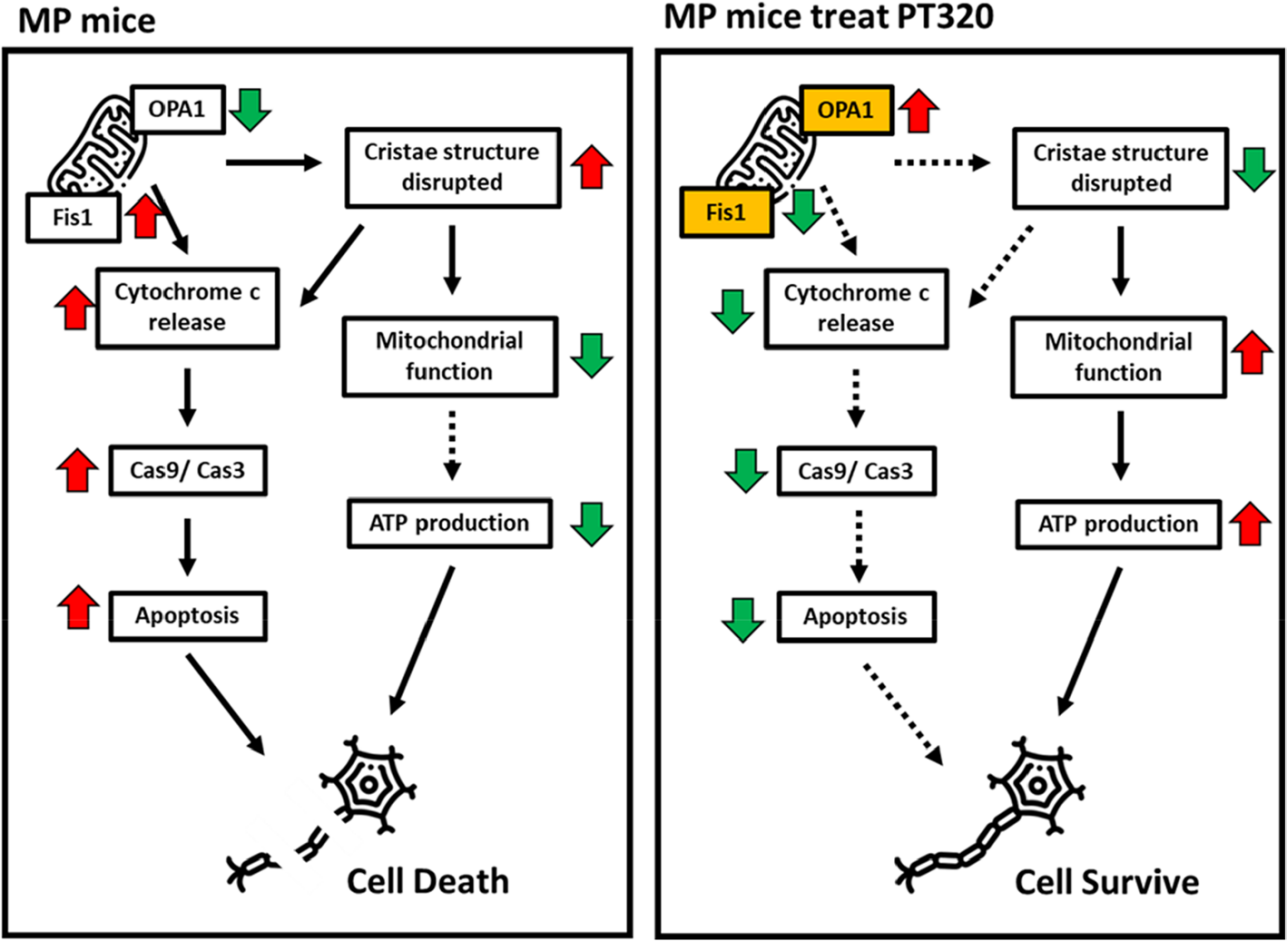
In summary, our findings suggest that PT320 regulates mitochondrial morphology by promoting Opa1 expression and inhibiting Fis1 expression during the early stage. Additionally, we observed that Opa1 has an influence on the release of cytochrome c, which is an upstream factor in apoptosis, by regulating the structure of mitochondrial cristae

In conclusion, our study demonstrated that the administration of a clinically translatable dose of Exenatide in the form of its clinical grade sustained release formulation PT320 delays the PD-like phenotype progression in MP mice. Notably, PT320 administration delayed the loss of TH expression. Additionally, PT320 reduced the levels of ROS, and inhibited the release of mitochondrial cytochrome c. Moreover, PT320 significantly prevented mitochondrial dysfunction and disruption of mitochondrial morphology. Although PT320 prevented mitochondrial damage, it did not affect the reduction in mitochondria number. Genetic analysis of the early disease stage revealed that PT320 regulates the expression of *Opa1* and *Fis1* genes. These genes play crucial roles in controlling mitochondrial morphology. *Opa1*, in particular, is involved in maintaining mitochondrial homeostasis and also inhibits cytochrome c release by remodeling the cristae of the mitochondria as well as thereby reducing apoptosis and tissue damage [29]. This is diagrammatically illustrated in Fig. 9.

Based on these findings, our study concluded that early treatment with PT320 may offer a promising neuroprotective therapy for patients with PD by preserving mitochondrial function. By targeting mitochondrial health and *Opa1* and *Fis1* regulation, PT320 holds promise as a novel future therapeutic approach for PD patients.

### Supplementary Information

**Supplementary Material 1.**

## Data Availability

The datasets used and/or analyzed during the current study are available from the corresponding authors on reasonable request.

## Abbreviations

GLP-1R: Glucagon-like peptide-1 receptor
PD: Parkinson's disease
ROS: Reactive oxygen species
Fis1: Mitochondrial fission protein 1
Opa1: Optic atrophy type 1
SNc: Substantia nigra pars compacta
ETC: Electron transport chain
Tfam: Mitochondrial transcription factor A
mtDNA: Mitochondrial DNA
MP: MitoPark
T2DM: Type 2 diabetes mellitus
TH: Tyrosine hydroxylase
DAT: Dopamine transporter
NDMC: National Defense Medical Center
NIDA: National Institute on Drug Abuse
NIH: National Institute of Health
PVDF: Polyvinyl difluoride
RT: Room temperature
TBST: Tris-buffered saline with Tween 20
PFA: Paraformaldehyde
OCT: Optimal cutting temperature
DAPI: Dye 4',6-diamidino-2-phenylindole
aCSF: Artificial cerebrospinal fluid
DCFDA: Dichlorodihydro fluorescein diacetate
TEM: Transmission electronic microscopy
OCR: Oxygen consumption rate
FCCP: Carbonyl cyanide-4-(trifluoromethoxy) phenylhydrazone
ANOVA: Analysis of variance
OXPHOS: Oxidative phosphorylation
Ex-4: Exenatide
6-OHDA: 6-Hydroxydopamine
NGS: Next generation sequencing
nDNA: Nuclear DNA
PCA: Principal component analysis
MQC: Mitochondrial quality control
MPTP: 1-Methyl-4phenyl-1,2,3,6-tetrahydropyridine
