# Supplementary material for: Attenuating mitochondrial dysfunction and morphological disruption with PT320 delays dopamine degeneration in MitoPark mice
Source: J Biomed Sci. 2024 Apr 17;31:38. doi: 10.1186/s12929-024-01025-6 (PMC11022395; doi:10.1186/s12929-024-01025-6)

**SUPPLEMENTARY FIGURES:**


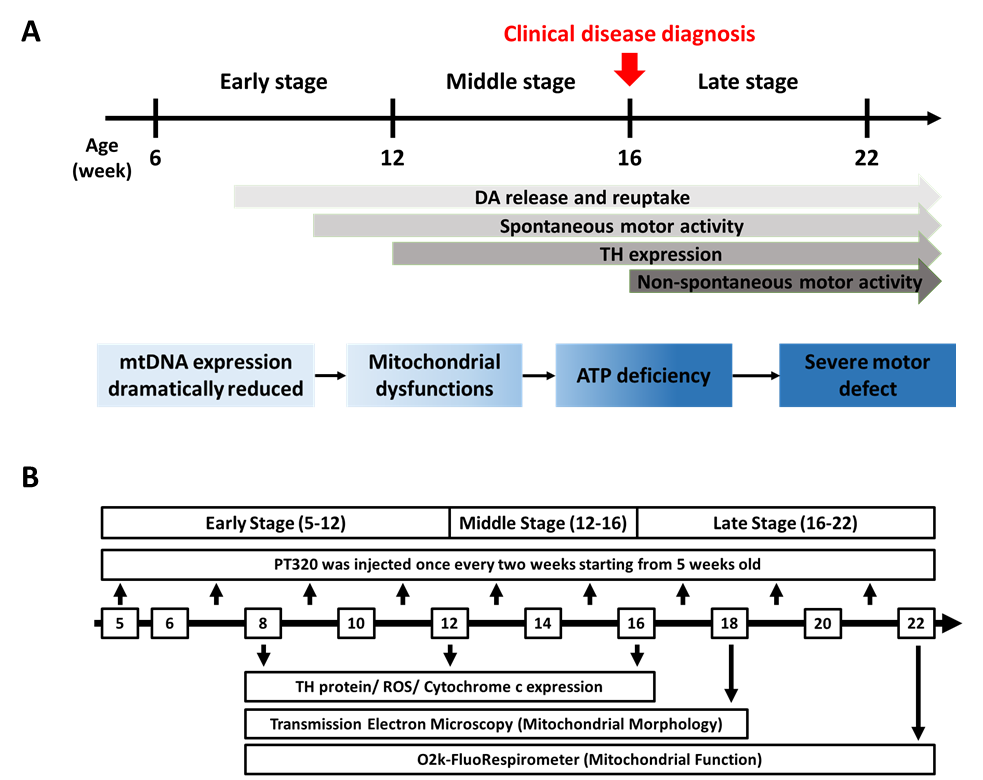


**Supplementary Fig. 1** (A) Similar to the disease course of PD in patients we divided the MP mice’ PD-like progressive phenotype into three stages. The early stage is the preclinical stage with no obvious phenotype characteristics. In the middle stage, the changes in DAN protein expression and neural function starts to decline, and the mice start to have small motor defects. In the late stage, mice show severe motor defects and significant dopaminergic neuron loss. (B) Schematic time line of biweekly administration of PT320 to MP animals and cellular as well as molecular tests.

**
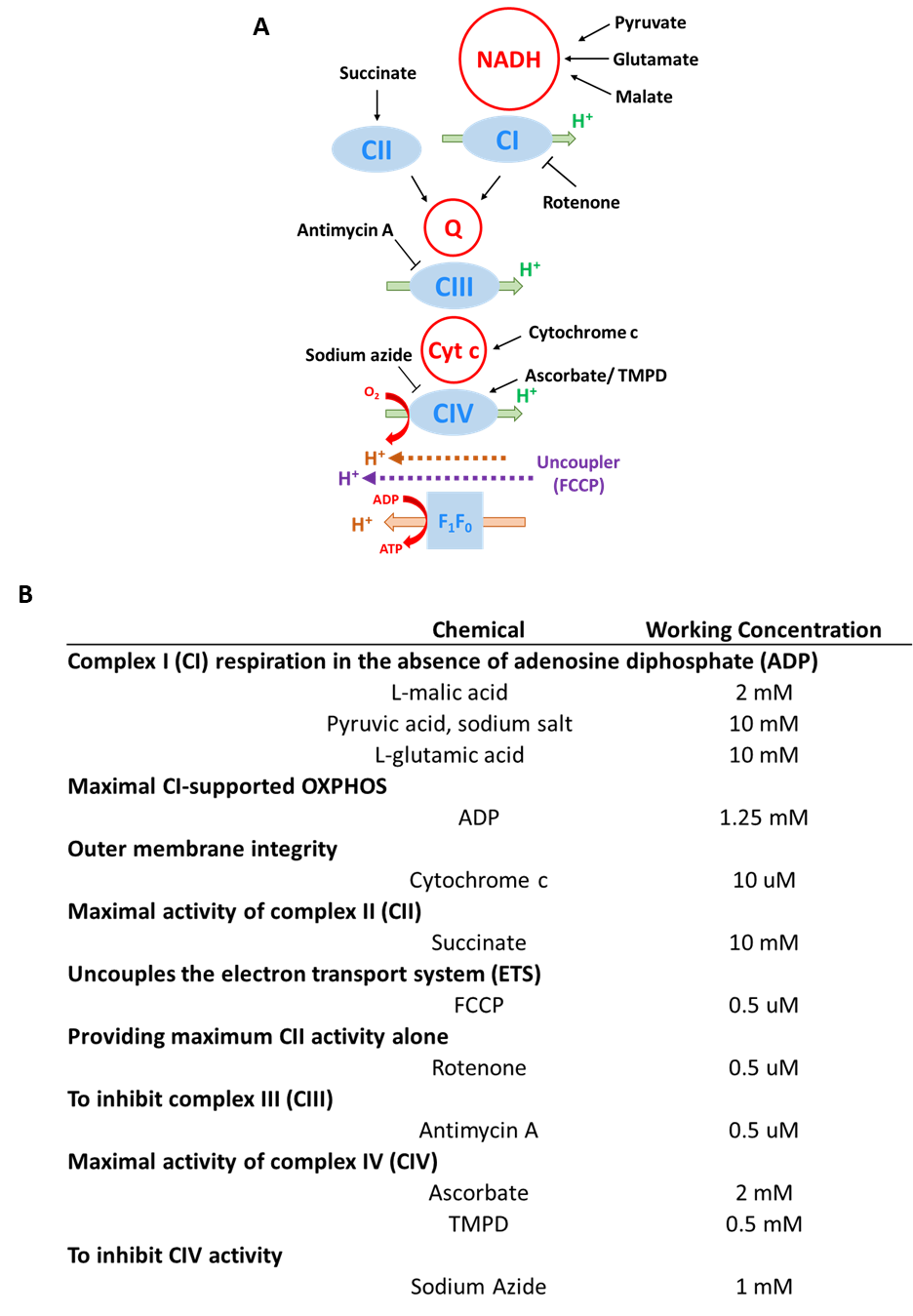
**

**Supplementary Fig. 2** The chemical reagents used in Oroboros O2k analysis and their diagrammed functions (A) as well as concentrations (B).


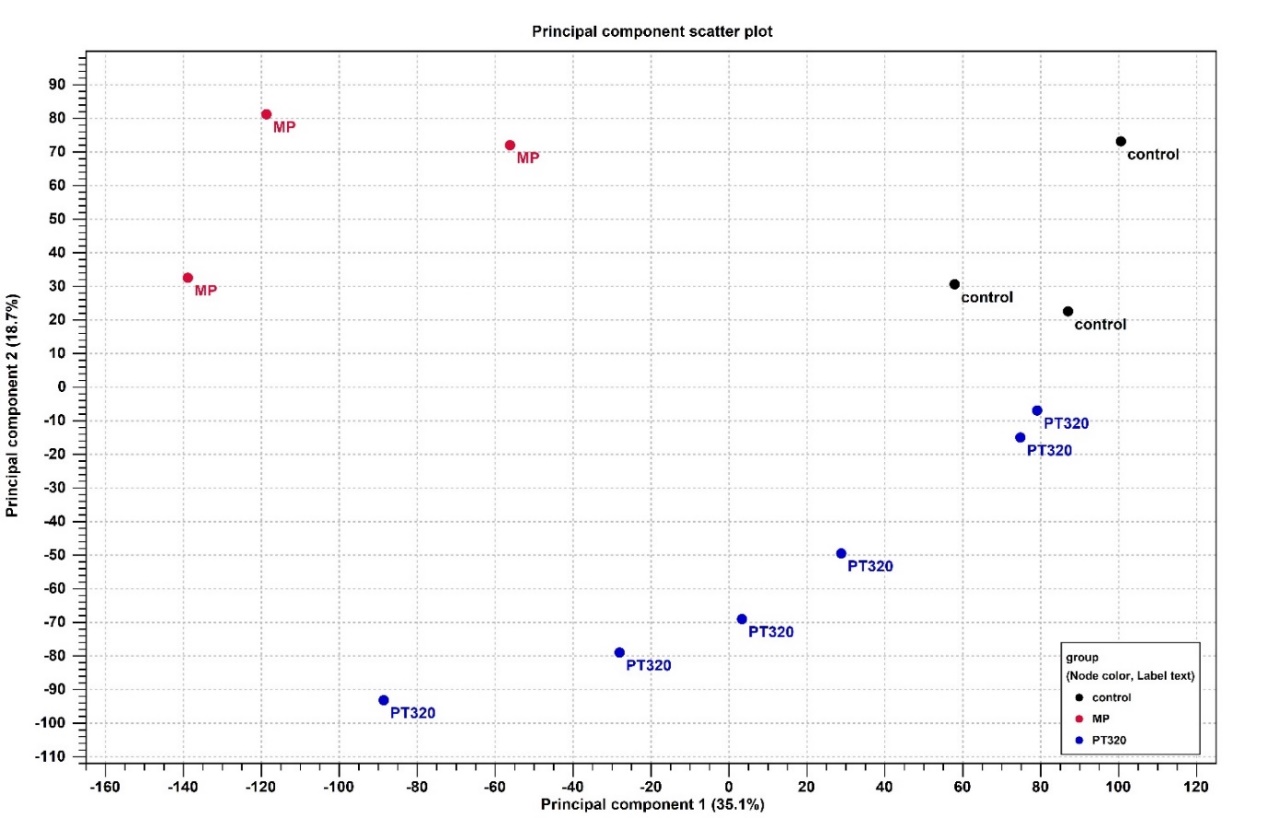


**Supplementary Fig. 3** Principle component analysis (PCA) of the indicated samples. The figure shows the genes’ distribution in samples; we used NGS analysis. The black (control) dots represent the WT mice, which are close together. The red (MP) dots are far from the control while the blue (PT320) dots are in a linear distribution and close to the control dots. This data indicates that PT320 administration affects the gene distribution in MP mice.


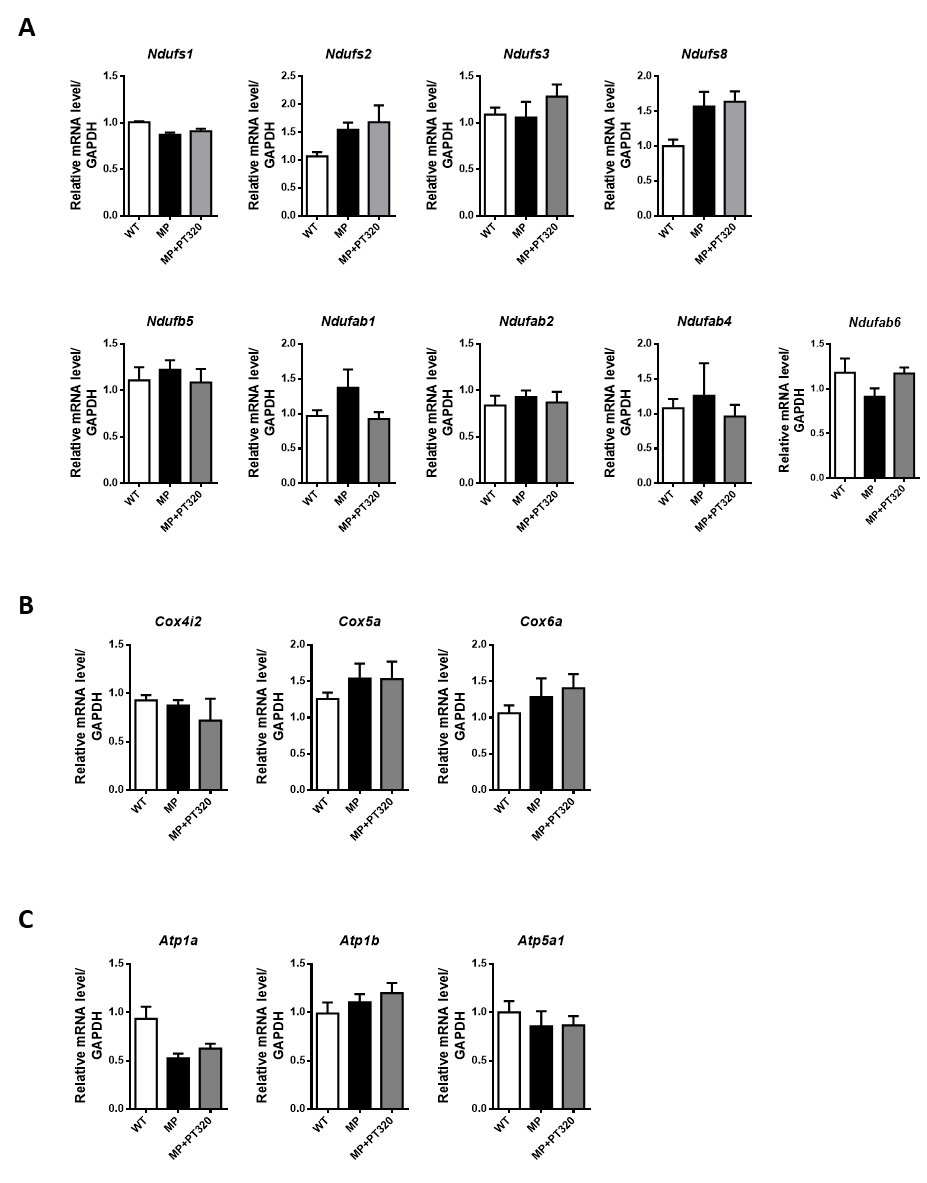


**Supplementary Fig. 4** Using qPCR, the expression levels of genes associated with complex I, IV, and V in NGS were confirmed, revealing FDR p-values < 0.05. However, qPCR analysis demonstrated no significant differences in the expression of these genes. This suggests that the regulation of mitochondrial function by PT320 may not occur directly through the modulation of complex-associated genes. (A) complex I associated genes. (B) complex IV associated genes. (C) complex V associated genes. (N = 6).


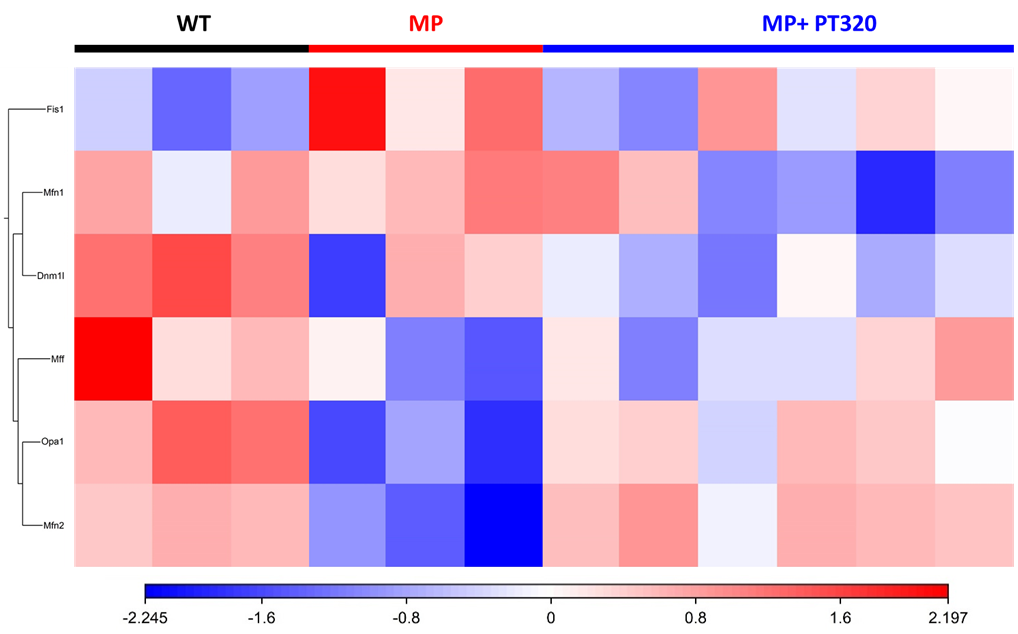


**Supplementary Fig. 5** The heat map of mitochondrial morphology-related gene expression in NGS data.


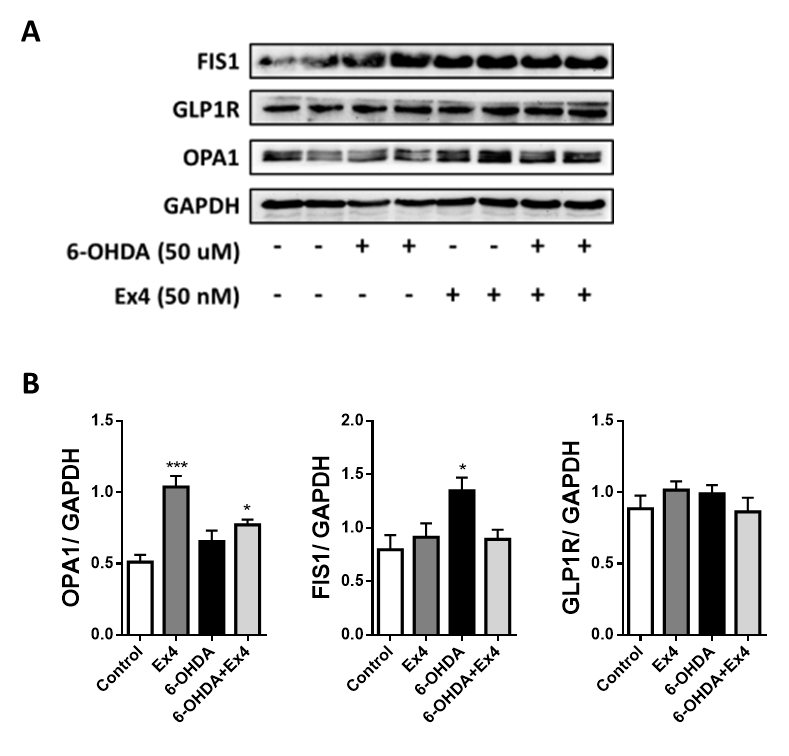


**Supplementary Fig. 6** Protein expression of GLP1R, FIS1, and OPA1 in N27 cells after 6-OHDA damage and Ex4 treatment. A) Western blots, B) Bar graphs of data and statistics. One-way analysis of variance (ANOVA) followed by Bonferroni post hoc test for multiple comparisons. WT vs MP or MP+PT320: *, p < 0.01; ***, p < 0.001.

**Supplementary Table 1** Primers used for mitochondrial complex I-V and mitochondrial morphology control genes.


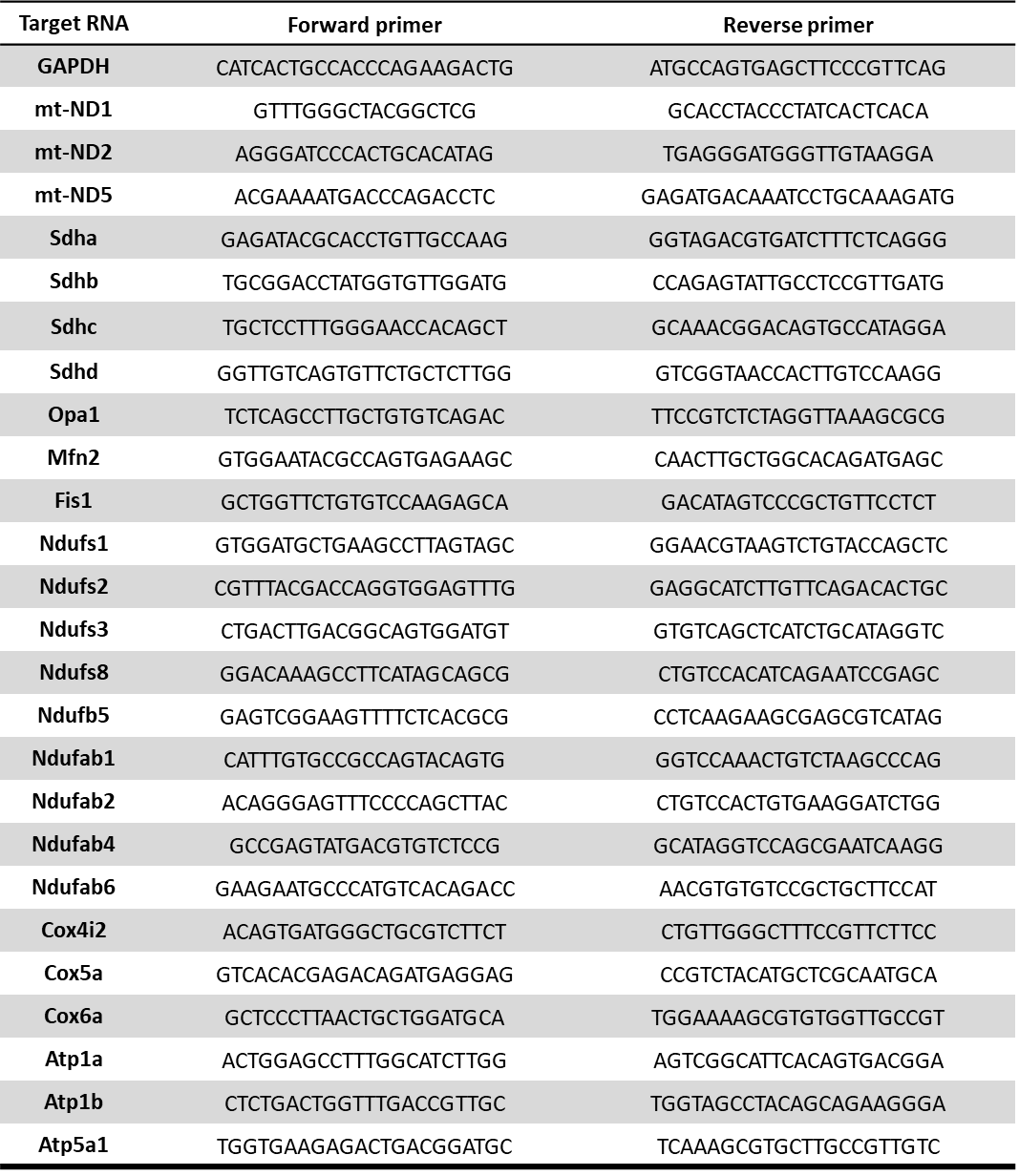

Supplement: Supplementary file 1 — Supplementary Material 1. [file 12929_2024_1025_MOESM1_ESM.docx]
